# Development and validation of a Chinese insulin medication literacy scale for patients with diabetes mellitus

**DOI:** 10.3389/fphar.2025.1477050

**Published:** 2025-04-02

**Authors:** Fangying Si, Tao Feng, Xiangfen Shi, Sufang Chen

**Affiliations:** ^1^ Department of Pharmacy, the First Affiliated Hospital of Zhengzhou University, Zhengzhou, China; ^2^ Department of Geriatric Endocrinology, the First Affiliated Hospital of Zhengzhou University, Zhengzhou, China

**Keywords:** insulin, medication literacy, diabetes mellitus, scale, reliability, validity

## Abstract

**Objective:**

This study aimed to develop a simplified insulin medication literacy scale for patients with diabetes mellitus in China (Ch-InMLS), assess the level of insulin medication literacy, and evaluate its psychometric properties.

**Methods:**

We developed an initial scale based on the framework of the knowledge–attitude–practice model (KAP), with “skills” added. The items were developed from literature review and insulin-related guidelines, semi-structured interviews, and face validity. After two rounds of expert consultation and a pilot survey, a final version of the scale was developed. A cross-sectional survey was conducted with 553 patients with diabetes mellitus in Zhengzhou for psychometric evaluation. In the construct validity analysis, the number of participants was 262 for exploratory factor analysis and 291 for confirmatory factor analysis. In the reliability analysis, internal consistency reliability and split-half reliability were evaluated using Cronbach’s alpha coefficients.

**Results:**

The final scale consists of 36 items with four domains: knowledge, attitude, practice, and skill. Exploratory factor analysis suggested four factors to explain 67.556% of the total variance (Kaiser–Meyer–Olkin test = 0.944, Bartlett’s test χ2 = 7384.296, *P* < 0.001). The results of confirmatory factor analysis showed that the model fits the data adequately. Cronbach’s coefficient was 0.945 for the overall scale, and for each domain, it was 0.952, 0.947, 0.908, and 0.923. The Spearman–Brown split-half reliability coefficient was 0.803 for the total scale, and for each domain, it was 0.925, 0.944, 0.901, and 0.917. The test–retest reliability coefficient of the total scale was 0.944, and for each domain of the scale, it was 0.865, 0.845, 0.987, and 0.936.

**Conclusion:**

The scale has acceptable content validity, construct validity, and good reliability. It can be used to evaluate the level of insulin medication literacy of patients with diabetes mellitus in China.

## 1 Introduction

In recent decades, the prevalence of diabetes mellitus (DM) has increased significantly, mainly as a result of a continuous rise in the incidence of type 2 DM. According to World Health Organization statistics, >422 million adults globally suffered from DM in 2014, and a continuous rise in DM prevalence is expected (Lovic et al., 2020). China is the most populous country with patients with diabetes: the prevalence of adult diabetes is 11.6% and prediabetes is 50.1%, according to an epidemiological study (Wang et al., 2021). The comprehensive control rate of DM was 2.0% (Ma et al., 2020), and only 39.7% of the estimated 113.9 million Chinese adults with diabetes have HbA1c ≤7.0% (Xu et al., 2013). This not only represents a heavy burden but also results in serious complications, such as diabetic nephropathy, diabetic retinopathy, and increased cardiovascular mortality (Cloete, 2022).

Intensive insulin treatment can help delay the onset of diabetes-related complications, and many patients with type 2 diabetes require insulin therapy at some stage to achieve or maintain good glycemic control (Riddle, 2021). However, many patients are still reluctant to initiate or adhere to insulin therapy due to reasons which include the fear of addiction, injection, side effects such as hypoglycemia and weight gain, inconvenience, and social stigma (Lee and Yoon, 2021; Dabas et al., 2023; Skriver et al., 2023). A systematic review (Boonpattharatthiti et al., 2024) indicated that the overall prevalence of adherence to insulin therapy is remarkably low, with adherence for T1D being 52.63% for T2D being 52.55%.

“Medication literacy” is the degree to which individuals can obtain, comprehend, communicate, and process patient-specific information about their medications in order to make informed decisions safely and to effectively use their medications, regardless of the mode by which the content is delivered (e.g., written, oral, and visual) (Pouliot et al., 2018). On this basis, Neiva Pantuzza et al. (2022) proposed a conceptual model for medication literacy which consisted of four domains: functional literacy, communicative literacy, critical literacy, and numeracy, including subdomains of accessing, understanding, evaluating, calculating, and communicating medication-related information. Compared to health literacy, “medications literacy” involves specific skills that are not completely covered in health literacy, such as understanding dosage instructions or information about a drug’s indication or adverse reaction. Medication literacy is thus a specialization of health literacy in the field of medicine.

Oral medications and insulin are effective treatments for type 2 diabetes, but insulin therapy is eventually indicated for patients once maximal doses of oral medications are no longer sufficient to control blood glucose levels. In type 1 diabetes, there is an absolute deficiency of insulin; insulin treatment should start from diagnosis for optimal glucose control and maintaining HbA1c. Several studies had shown that reasons for low adherence to insulin therapy are public embarrassment (Farsaei et al., 2014; Peyrot et al., 2012), concern over hypoglycemia (Peyrot et al., 2012; Bermeo-Cabrera et al., 2018), negative beliefs and attitude about insulin therapy (Yavuz et al., 2015), difficulties in preparing injection, poor knowledge regarding DM, and insulin self-injection (Mariye et al., 2019).Therefore, the systematic identification of people with limited ability to take responsibility for their insulin therapy is critical. To the best of our knowledge, most of the instruments available regarding insulin do not cover essential aspects of insulin literacy such as correct thinking, favorable attitude, and the ability to access and information on insulin. A structured and regular assessment of patients’ insulin literacy should be conducted to ensure that they are managing their insulin appropriately. This is especially necessary for those with type 1 diabetes who must use insulin therapy as their mainstay and type 2 diabetes who cannot control or tolerate oral medications. Until now, there have been a variety of generic medication literacy scales for the general population and particular populations or diseases. There are also some medication literacy scales for particular drugs (Table 1). Nonetheless, there is no specific scale to assess insulin medication literacy for patients with DM.

**TABLE 1:** Summary of generic medication literacy scales for general population, particular population or disease, and particular drug.

NO	Authors	Scope of application	Scale title
1	Emmerton et al. (2012)	General population	Health literacy of pharmacy consumer questionnaire
2	Zhong et al. (2020a)	General population	Medication literacy questionnaire for discharged patients
3	Sauceda et al. (2012)	General population	Medication literacy in Spanish and English assessment tool
4	Stilley et al. (2014)	General population	Medication health literacy measure
5	Vervloet et al. (2018)	General population	Recognizing and addressing limited pharmaceutical literacy interview guide
6	Yeh et al. (2017)	General population	Chinese medication literacy measure
7	Zhang et al. (2021)	Particular population or disease	Pregnant women’s medication information literacy scale
8	Zhong et al. (2020b)	Particular population or disease	Chinese medication literacy scale for hypertensive patients
9	Ubavić et al. (2018)	Particular population or disease	Pharmacotherapy literacy questionnaire for patients of pre-school children
10	Pantuzza et al. (2023)	Particular population or disease	Medication literacy test for older adults (TELUMI)
11	Gnägi et al. (2022)	Particular population or disease	Medication literacy assessment instrument (MELIA) for older people receiving home care
12	Jang et al. (2019)	Particular drug	Medication label literacy instrument focused on non-steroidal anti-inflammatory drugs
13	Shreffler-Grant et al. (2014)	Particular drug	Complementary and alternative medicine health literacy scale

This scale was developed based on the conceptual framework of the knowledge–attitude–practice model (KAP) (Cleland, 1973), with “skills” added in the context of medication use. The framework describes four main competencies for making informed insulin related decisions: knowledge, attitude, practice, and skill. The aim of this study was to develop a valid and reliable assessment scale for use by medical staff and evaluate its psychometric properties for patients with DM in China.

## 2 Materials and methods

### 2.1 Study setting and design

This research was conducted during a cross-sectional, multi-center survey of the qualitative study. Data collection was carried out from April 2024 to June 2024, and all participants completed an informed consent.

**FIGURE 1:**
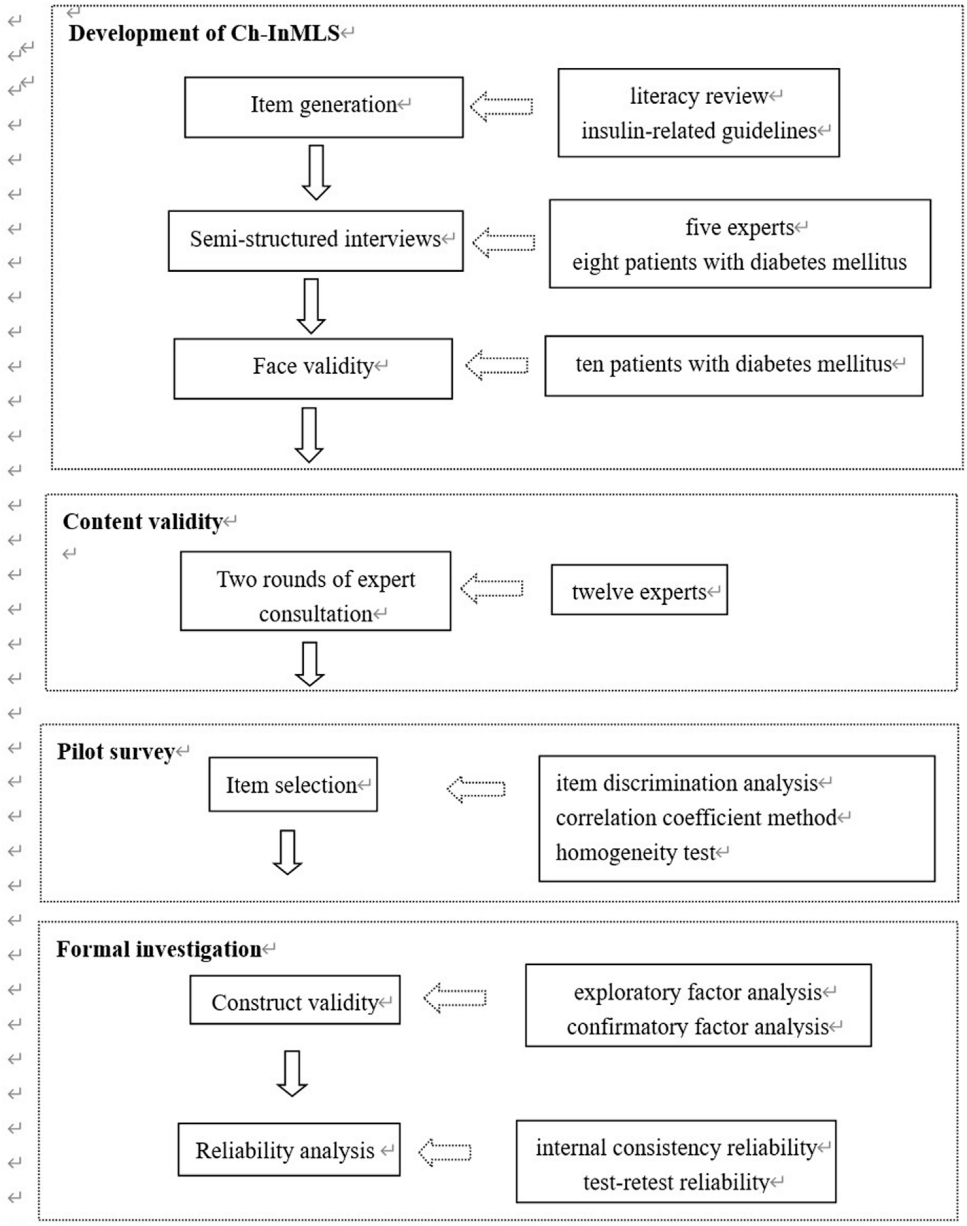
Process of scale development validation.

The study was carried out in four phases. In the first phase, the Chinese Insulin Medication Literacy Scale (Ch-InMLS) for patients with DM pool items were developed. In the second phase, content validity was conducted by 12 experts working in DM management or care, who were invited for a two-round Delphi (Jünger et al., 2017). In the third phase, 200 patients with DM were tested in the pilot study, and the items were revised or deleted by item discrimination analysis, the correlation coefficient method, and a homogeneity test. In the fourth phase, formal investigation was conducted among 553 patients with DM by construct validity and reliability analysis. Figure 1 shows the process of questionnaire development.

### 2.2 Participants

Participants were recruited by convenience sampling at outpatient clinics and wards of the First Affiliated Hospital of Zhengzhou University, which is a Grade-A tertiary general hospital tertiary hospital, and two community health service centers in the city of Zhengzhou in China. The investigations of participants were conducted independently in three phases using the online platform Questionnaire Star (Changsha Ranxing Information Technology Co., Ltd.), a free questionnaire platform widely used in China. The first stage was pilot testing which included 200 participants. The second stage was the formal investigation with 553 participants. The third stage was the retest survey of 40 participants collected from the formal investigation by simple random sampling. The inclusion criteria of the experts who joined in the semi-structured interviews and two-round Delphi were: (1) over 8 years practical experience of DM management or care; (2) intermediate or senior titles; (3) specialized in the development and psychometric validation of a scale; (4) willing to participate in our study. The experts were recruited by snowball sampling from the First Affiliated Hospital of Zhengzhou University, the Second Affiliated Hospital of Zhengzhou University, and Henan Provincial People’s Hospital. The inclusion criteria of the participants were as follows: (1) diagnosed with type 1 or 2 diabetes according to the 1999 WHO diagnostic criteria for diabetes; (2) aged over 18 years; (3) the ability to read and write or use WeChat; (4) have been under insulin treatment for at least 4 weeks and should be under insulin treatment perpetually, included both those newly diagnosed and being treated with insulin for a short period and those who already under insulin treatment (once or more per day) for a longer period of time. The exclusion criteria included the following: (1) patients with a history of cognitive impairment or psychiatric disease; (2) patients who declined participation; (3) patients with hearing and communication disability.

### 2.3 Phase 1: Development of Ch-InMLS

The questionnaire’s development consisted of the following steps: item generation, semi-structured interviews, and face validity.

### 2.4 Item generation

**FIGURE 2:**
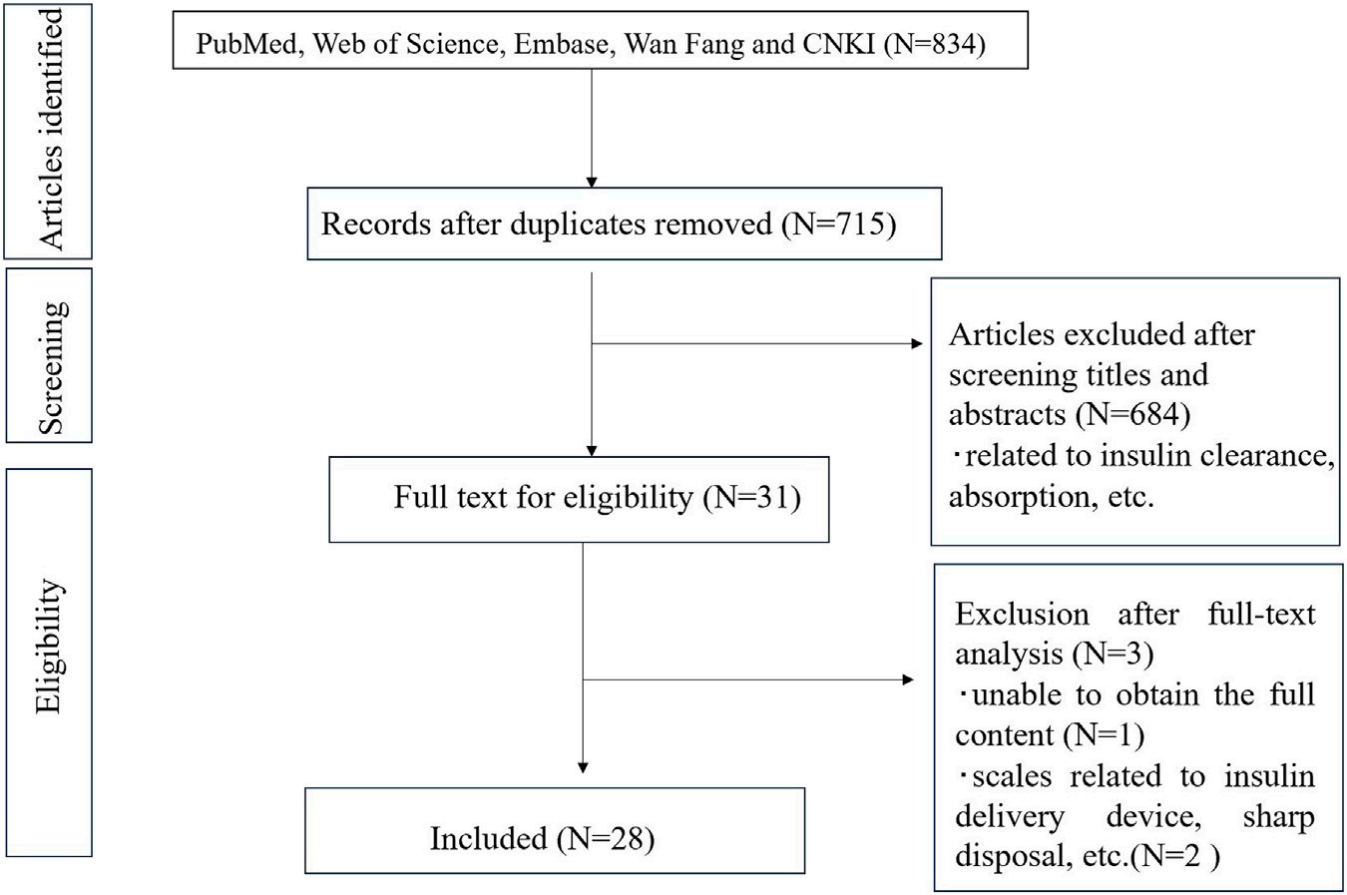
Flow diagram of literature review.

For the present study, we conducted a comprehensive literature review from electronic databases including PubMed, Web of Science, Embase, Wan Fang, and CNKI Data relating to the insulin medication literacy of patients with DM without time restrictions. A search strategy was developed, combining the following keywords: (insulin) AND (scale OR measurement OR questionnaire OR tool). The inclusion criteria for the articles were: (1) relevant to the development, revision and psychological measurement of insulin-related scales; (2) quantitative and/or qualitative studies; (3) published in English or Chinese domain. The complete search process is available in Figure 2.

A deductive method was conducted to generate items (Elo and Kyngäs, 2008) related to each domain (knowledge, attitude, practice, and skill) and enrich the composition of Ch-InMLS. We reviewed and evaluated current literature and insulin-related guidelines to generate items. For example, items extracted from scales assessing psychology status could be classified as “knowledge” or “attitude”, and items extracted from scales assessing self-management and guidelines could be classified as “practice” or “skill” (Table 2). Duplicate items were subsequently removed. Soon afterward, an initial draft was developed in English, subsequently translated to Chinese using the back-translation method.

**TABLE 2:** Example of implementation of the deductive method.

Sources of items	Item	Domain
Literatures/guidelines		
Snoek et al. (2007)	Insulin will make others perceive greater sickness.	Knowledge
Snoek et al. (2007)	Insulin signifies failure with pre-insulin therapy.	Knowledge
……	……	……
Fu et al. (2013)	I worry that people will know I have diabetes if I am on insulin treatment.	Attitude
Fu et al. (2013)	Injecting insulin is embarrassing, I worry about being seen when I inject insulin.	Attitude
……	……	……
Karahan Okuroglu et al. (2020)	I can adjust my insulin dose according to my blood sugar.	Practice
Expert consensus on insulin in primary care for type 2 diabetes, 2021	Patients were advised to monitor their blood glucose.	Practice
……	……	……
Karahan Okuroglu et al. (2020)	I regularly change the sites where I inject insulin.	Skill
Expert consensus on insulin in primary care for type 2 diabetes,2021	After completion of the insulin bolus, I keep the needle in place for at least 10 s before pulling it out.	Skill
……	……	……

### 2.5 Semi-structured interviews

The researchers contacted those interviewed directly by phone. The time and place of the interview were arranged according to the convenience of each participant. The interviews were conducted by researcher SFY and recorded by researchers FT and SXF. The entire interview process was audio-recorded. Data saturation was defined as “no new themes or codes emerging from interviews.” The questions asked to the experts and the patients with DM are shown in Table 3. Inclusion criteria for patients enrolled in this stage were the same as the participants criteria mentioned above. Interview data were analyzed using thematic analysis, a method widely used in qualitative research to identify, analyze, and report data patterns. The topics extracted from the interview data were developed into items based on the four core elements of insulin literacy for DM patients: knowledge, attitude, practice, and skill.

**TABLE 3:** Questions used in semi-structured interviews.

For experts
1. What do you think is significant in insulin self-management in clinical practice?
2. What misunderstandings do you find in insulin therapy for diabetics?
3. Share the most impressive experience you had about insulin management

For patients with diabetes mellitus
1. Do you have any strengths with insulin use and what are they?
2. Do you have any barriers to insulin use and what are they?
3. What would you expect to know about insulin from your health care provider?
4. Share the good and bad experiences you have had about insulin.

At this stage, an inductive method was used and combined with the above deductive method to develop an initial draft scale.

### 2.6 Face validity

Face validity of the initial drafted scale was conducted with ten DM patients through face-to-face individual interviews. The patients were invited to check the readability, comprehensibility, and response errors of the draft scale. Feedback and advice as well as questions proposed by interviewed patients on each item were recorded, and complex items with technical words which were hard to understand were replaced by more popular terms. The researchers then communicated with the participants and formulated an original scale based on the participants’ feedback and advice.

### 2.7 Phase 2: Content validity

A panel of 12 experts was invited to appraise the construct and 37 items of the primary insulin literacy scale in this study. We conducted a two-round Delphi by sending emails to the experts. The questionnaire was composed of three parts. The first part collected general information about the experts, including age, work experience, educational background, and professional title. The second part included the expert’s familiarity degree with the survey content. The third part involved scoring the importance of self-assessment tool indicators using a Likert 5-point scale, ranging from 1 (not important at all) to 5 (very important), with an additional recommendation column. Experts assessed expression, grammar, phrasing, and item allocation of the scale according to their comprehension of the connotations of insulin medication literacy, and their suggestions and the rationales were encouraged. The first-round questionnaire results were analyzed and fed back to respondents before the second round of consultation. Expurgations and revisions of items or contents were made at the end of the first round, generating the second round of the questionnaire. Indexes not gaining consensus in the first round were repeated in the subsequent survey until consensus was reached, and the index system was constructed.

The authority coefficient of experts was computed by their familiarity degree with the concept of insulin medication literacy and judgmental reference. The formula used for authority coefficient (Cr) calculation was Cr = (Ca + Cs)/2, with Ca representing experts’ judgment criteria and Cs representing the degree of their familiarity with each indicator. The judgment criteria were based on four aspects: theoretical analysis, practical experience, reference literature at home and abroad, and intuitive feeling (Table 4). For the assigned Cs scores, 1 point was for more familiar, 0.8 points for familiar, 0.5 points for general, 0.2 points for unfamiliar, and 0 points for ignorant. An authority coefficient (Cr) of over 0.8 was considered acceptable (McPherson et al., 2018).

**TABLE 4:** Judgment criteria of experts.

Judgment basis	Degree of contribution to expert judgment		
	Large	Medium	Small
Theoretical analysis	0.3	0.2	0.1
Practical experience	0.5	0.4	0.3
Reference literature at home and abroad	0.1	0.1	0.1
Intuitive feeling	0.1	0.1	0.1

For the expert coordination coefficient, Kendall’s coefficients of concordance (Kendall’s W) ranged from 0.40 to 0.59, indicating a grudgingly acceptable degree of chance agreement (Chen et al., 2022). Content validity was assessed by calculating a content validity index (CVI) for the overall scale (S-CVI/Ave) and for each item of the scale (I-CVI). The CVI was assessed by asking the experts to rate each item according to the item’s relevance on a four-point scale: 1 = not relevant, 2 = slight relevance, 3 = certain relevance, and 4 = very relevant. A CVI score of 0.79 or above for each item was considered acceptable. Items with a CVI score between 0.70 and 0.79 were revised and those with a CVI score less than 0.70 were excluded (Lynn, 1986; Polit et al., 2007)

### 2.8 Phase 3: Pilot survey

To reduce the number of items, the 37-item Ch-InMLS was pre-tested in a total of 220 patients with DM. At this stage, a total of 200 questionnaires were received back and checked for validity. The response rate was 90.91%. The statistical analysis methods used for item selection were as follows.

*Item discrimination analysis*. Total scores of collected questionnaires were ranked from high to low, of which 27% with low total scores were considered the low score group, and 27% with high total scores were considered the high score group. Each item in two groups was tested for difference using independent t-testing, and items with no significant difference in scores between the two groups were excluded (*P* > 0.05) (Kelley, 1939; Metsmuuronen, 2020).

*Correlation coefficient method*. Pearson’s correlation coefficients between each item and the overall scale, and between each item and its belonging domain, were calculated. Items with a Pearson’s correlation coefficient *r* <0.4 were considered low correlation and were recommended for removal (Wu, 2010).

*Homogeneity test*. If an item was deleted and a significant increase was present in the alpha coefficient, then deletion was considered. Communalities of less than 0.2 were also considered for removal (Wu, 2010).

### 2.9 Phase 4: Formal investigation

To examine the psychometric properties of the tool, construct validity, criterion validity, and reliability were assessed.

The construct validity of this scale was assessed by exploratory factor analysis (EFA), which is generally used to generate the factor structure and confirmatory factor analysis (CFA) to test the fit of the hypothetical factor structure. EFA was conducted to extract factors by performing principal components analysis with the maximum variation method. The Kaiser–Meyer–Olkin (KMO) coefficient and Bartlett’s sphericity test were used to assess the suitability of the data. The factor structure obtained from EFA was then tested by CFA. Goodness-of-fit was evaluated by using the chi-square minimum/degree of freedom (χ2/df), root mean square error of approximation (RMSEA), goodness of fit index (GFI), comparative fit index (CFI), incremental fit index (IFI), parsimonious normed fit index (PNFI), and parsimonious goodness-of-fit index (PGFI). The reasonable threshold levels of these indices for CFA were considered χ 2/df <3 (Hu and Bentler, 1998), for RMSEA a value <0.08 (Pett et al., 2003; Sun, 2005; Hair et al., 2009), for GFI, AGFI, and IFI >0.90, RMR <0.05 (Pett et al., 2003; Hair et al., 2009), and PNFI and PGFI >0.50 (Mulaik et al., 1989).

The convergent and discriminant validity of the scale were assessed, and standardized factor loadings, average variance extracted (AVE), and composite reliability (CR) were calculated for the final model. Convergent validity evaluates the degree of correlation of multiple items of the same domain both theoretically and practically. AVE >0.5 and CR >0.7 confirmed that convergent validity was satisfying. Discriminant validity indicates the level of difference between different latent variables and is valid if the average variance is greater than squared correlation coefficients (Chen et al., 2023).

The reliability validity of this scale was assessed by internal consistency and stability. Cronbach’s alpha value was calculated to evaluate the internal consistency of the total scale and each domain, and a value >0.7 was satisfactory and considered as good internal reliability (Liu and Liu, 2010). Test–retest reliability was used to evaluate the stability of the scale, and it was measured by Pearson’s correlation coefficient in 40 randomly collected participants from the 553 patients 2 weeks after formal investigation. A value of correlation coefficient over time >0.75 (*P* < 0.05) was considered good test–retest reliability (Koo and Li, 2016).

For scoring criteria for Ch-InMLS, for items K6 to K10, P5, and S2, answering right for each item scores 2, and answering wrong or “I don’t know” scores 1. For items K1 to K5 and A1 to A11, the response option of the 5-point Likert scale (totally agree, agree, not sure, disagree, and totally disagree) for each item, scores of 5, 4, 3, 2, and 1 were applied accordingly. For items P1 to P4, P6, P7, S1, and S3 to S8, the response option of 5-point Likert scale (always, often, sometimes, seldom, and never) for each item, scores of 5, 4, 3, 2, and 1 were also used accordingly. There were eight items in the attitude domain scoring reversely. The summed total score on this 37-item scale ranged from 37 to 164, with higher scores indicating a higher insulin medication literacy level.

We followed the Guidelines for Reporting Reliability and Agreement Studies (GRRAS) and The Quality Appraisal of Reliability Studies (QAREL) checklist for reporting (Supplementary Material S1).

## 3 Results

### 3.1 Development of the Ch-InMLS

A total of 40 items were screened by the literature review and insulin-related guidelines. After adding semi-structured interview results, four items were added. Subsequently, face validity was conducted among ten patients with diabetes mellitus (DM), and four items were revised. Until now, the item pool comprised 44 items (see Supplementary Material S2).

### 3.2 Content validity

At this stage, 12 experts were invited respectively for two rounds of Delphi consultations. After the first round, five items were deleted and two were revised. After the second round, two items were revised (see Supplementary Material S3). After the completion of the two Delphi rounds, the opinions of the experts were basically consistent, and a preliminary scale was finally developed. The positive coefficients of the experts were 100% and 100%, indicating that the experts in Delphi consultation were voluntary and active to participate to comment on the scale. The results of the expert consultation also showed that the individual authority coefficient of each expert ranged from 0.85 to 0.95 and the integrated authority coefficient of all experts was 0.89, meaning that the evaluation and recommendations generated from this expert panel were considerably authoritative and can be trusted. Furthermore, Kendall’s W was 0.416 (*P* < 0.05) and 0.582 (*P* < 0.05) for the two rounds, indicating a grudgingly acceptable degree of chance agreement on item appraisements. The I-CVI (item-level content validity index) of each item ranged 0.833 to 1.000, which were >0.79, and the S-CVI (scale-level content validity index) for the total scale was 0.935, indicating a good content validity for the scale.

### 3.3 Pilot survey

#### 3.3.1 Item discrimination analysis

The difference of each item between the high and low score groups was tested by independent t-test (Supplementary Material S4). Except for item A12, the difference between the high and low score group of the leftover 36 items were all significant (*P* < 0.001).

#### 3.3.2 Correlation coefficient method

For the knowledge domain, Pearson’s correlation coefficients between items and the domain ranged from 0.747 to 0.950 (*P* < 0.001). For the attitude domain, except for item A12 with a Pearson’s correlation coefficient of −0.064, Pearson’s correlation coefficients between other items and the domain ranged from 0.734 to 0.914 (*P* < 0.001). For the practice domain, Pearson’s correlation coefficients between each item and the domain ranged from 0.695 to 0.870 (*P* < 0.001). For the skill domain, Pearson’s correlation coefficients between each item and the domain ranged from 0.701 to 0.858 (*P* < 0.001). Pearson’s correlation coefficients between each item and the overall scale ranged from 0.406 to 0.688 (*P* < 0.001) —except for item A12 with a Pearson’s correlation coefficient of −0.130 (see Supplementary Materials S5–S9).

#### 3.3.3 Homogeneity test

Except for item A12, Cronbach’s α coefficient of the remaining 36 items after item deletion had decreased (Supplementary Material S10). Except for items A5, A10, and A12, the communalities of the remaining 34 items >0.2 (see Supplementary Material S11).

After comprehensive consideration, item A12 was considered for deletion, the formal insulin medication literacy scale for patients with DM had been fulfilled, and four domains with 36 items were confirmed.

### 3.4 Formal investigation

A total of 570 patients with DM participated in the questionnaire, of which 553 were collected—a response rate of 97.01%. The age of the participants ranged from 25 to 84, with a mean age of 62.60 (SD = 10.76). Of the 553 participants, 289 were men (52.3%), 151 had an education level of primary school or below (27.3%), 496 had been married (89.7%), 176 were employed (31.8%), 444 had been diagnosed with DM for more than 10 years (80.3%), and 167 participants (30.2%) had a family history of DM (Table 5).

**TABLE 5:** Basic demographic characteristics.

Items	Group	N	%
Age (years)^a^	18∼45	23	4.2
	46∼60	179	32.4
	>61	351	63.5
Gender	Male	289	52.3
	Female	264	47.7
Education level	Primary and below	151	27.3
	Junior middle school	197	35.6
	High school	136	24.6
	College degree and above	69	12.5
Marital status	Unmarried	22	4.0
	Married	496	89.7
	Divorced/widowed	35	6.3
Occupational status	Employed	176	31.8
	Unemployed/retired	377	68.2
Duration of diabetes	≤3 years	23	4.2
	4∼9 years	86	15.6
	≥10 years	444	80.3
Registered residence	Urban	325	58.8
	Countryside	228	41.2
Annual household income Chinese CNY (¥)	<10,000/year	43	7.8
	10,000∼29,999/year	235	42.5
	30,000∼49,999/year	154	27.8
	50,000∼99,999/year	121	21.9
	≧100,000/year		
Family history of diabetes mellitus	Yes	167	30.2
	No	386	69.8

^a^ Mean for age was 62.60 years with a standard deviation of 10.76.

#### 3.4.1 Construct validity

EFA was performed to identify the main factors of the tool. The construct and factor structure of this scale and of each domain were analyzed by principal component analysis with Varimax rotation. Some 262 collected questionnaires were randomly abstracted from the 553 collected to conduct the EFA for the scale.

Kaiser–Mayer–Olkin (KMO) measured for the overall scale indicated that the sample size for this scale was adequate with a value of 0.944, and Bartlett’s test of sphericity was significant (χ2 = 7384.296, *P* < 0.001). The percentage of the total variance was 67.556%, and four domains were extracted (Table 6). In each domain, rotation factor loadings of all items were greater than 0.6, and no item was loaded on more than one domain. The number of domains extracted by EFA and the items belonging to each domain were consistent with the initial conceptual framework. Therefore, four components of the overall scale were identified. Domain 1 was labelled “knowledge” and contained ten items; domain 2 was labelled “attitude” and contained eleven items; domain 3 was labelled “practice” and contained seven items; domain 4 was labelled “skill” and contained eight items.

**TABLE 6:** Exploratory factor analysis on the scale of Ch-InMLS for patients with diabetes mellitus (n = 262).

Item	Factors			
	1	2	3	4
K5	0.888			
K2	0.874			
K7	0.789			
K4	0.786			
K1	0.786			
K3	0.782			
K9	0.777			
K6	0.773			
K10	0.767			
K8	0.726			
A2		0.905		
A3		0.838		
A4		0.833		
A9		0.825		
A8		0.824		
A6		0.818		
A5		0.787		
A1		0.781		
A11		0.780		
A10		0.770		
A7		0.724		
P5			0.817	
P7			0.769	
P6			0.765	
P3			0.756	
P2			0.726	
P4			0.704	
P1			0.688	
S2				0.856
S1				0.788
S7				0.784
S3				0.766
S8				0.754
S4				0.752
S5				0.746
S6				0.664
Eigenvalues	6.216	12.359	2.638	3.108
Explained variations (%)	19.427	20.893	12.537	14.699
Total explained Variations (%)	67.556			

Note: K is short for “knowledge”, A for “attitude”, P for “practice”, and S for “skill”. KMO measure of sampling adequacy value = 0.944; Bartlett’s test: χ2 (chi square test value) = 7384.296; df (degree of freedom) = 630; P = 0.000.

**FIGURE 3:**
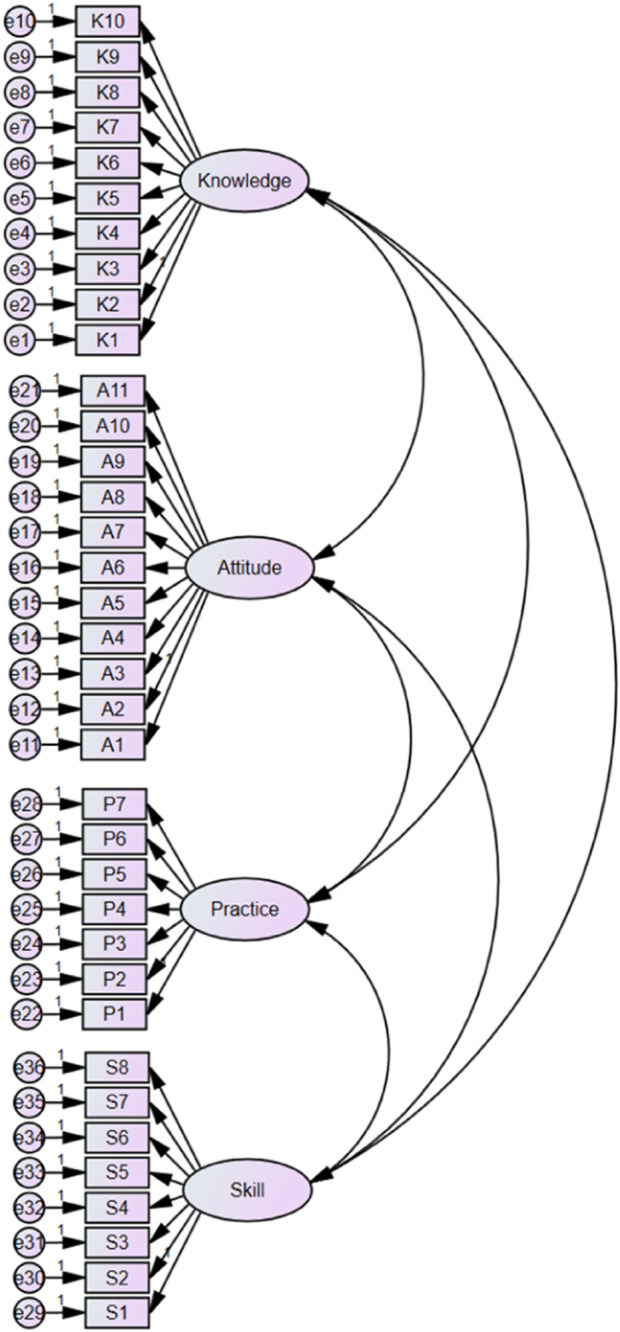
Structure equation modeling of four domains for insulin medication literacy scale. NB: K is short for “knowledge”, A for “attitude”, P for “practice”, and S for “skill”.

Based on the EFA results, the remaining 291 instruments of the data were used by confirmatory factor analysis (CFA) for formal comparison of models. Most fitting indexes of the four-factor models reach reference values, indicating that the model has a good degree of fit (Table 7). The results of structure equation modeling for the CFA of scale are shown in Figure 3.

**TABLE 7:** Results of fitting indices of confirmatory factor analysis of four-domain model of Ch-InMLS for diabetes mellitus (n = 291).

Parameters	Four-domain model	Reference value
χ^2^/df	1.237	≤3
GFI	0.878	>0.90
AGFI	0.862	>0.90
RMR	0.056	<0.05
IFI	0.982	>0.90
RMSEA	0.029	<0.08
PGFI	0.916	>0.50
PNFI	0.850	>0.50

Convergent validity analysis showed that the standardized factor loading values of each item ranged from 0.647 to 0.927. The CR values ranged from 0.911 to 0.951 and the AVE values from 0.596 to 0.661. The convergent validity was acceptable. The square roots of the AVE were greater than the correlations between the domains of the scale, indicating the reasonable discriminant validity of the scale (Table 8).

**TABLE 8:** Convergent and discriminant validity of the scale.

Total scale/Domains	Sted	Square correlation	CR	AVE	Square root of AVE
K1	0.862	0.743	0.951	0.661	0.813
K2	0.910	0.828			
K3	0.796	0.634			
K4	0.783	0.613			
K5	0.870	0.757			
K6	0.812	0.659			
K7	0.767	0.588			
K8	0.753	0.567			
K9	0.792	0.627			
K10	0.770	0.593			
A1	0.825	0.681	0.944	0.606	0.778
A2	0.893	0.797			
A3	0.751	0.564			
A4	0.817	0.667			
A5	0.647	0.419			
A6	0.795	0.632			
A7	0.666	0.444			
A8	0.790	0.624			
A9	0.769	0.591			
A10	0.754	0.569			
A11	0.820	0.672			
P1	0.663	0.440	0.911	0.596	0.772
P2	0.759	0.576			
P3	0.726	0.527			
P4	0.769	0.591			
P5	0.927	0.859			
P6	0.793	0.629			
P7	0.740	0.548			
S1	0.770	0.593	0.928	0.616	0.785
S2	0.859	0.738			
S3	0.767	0.588			
S4	0.678	0.460			
S5	0.815	0.664			
S6	0.809	0.654			
S7	0.822	0.676			
S8	0.746	0.557			

Note: K is short for “knowledge”, A for “attitude”, P for “practice”, and S for “skill”.

#### 3.4.2 Reliability analysis

The Cronbach’s α coefficient of the scale was calculated for internal consistency. The results showed that the Cronbach’s α coefficient for the overall scale of 36 items was 0.945, and Cronbach’s α coefficient for each domain was 0.952, 0.947, 0.908, and 0.923, respectively, indicating that internal consistency reliability of the scale was established. The total scale Spearman–Brown split-half correlation coefficient was 0.803, while that for each domain was 0.925, 0.944, 0.901, and 0.917, respectively. The retest reliability coefficient for the overall scale was 0.944, and for each domain of the scale was 0.865, 0.845, 0.987, 0.936 (*P* < 0.001), respectively, suggesting the consistency of the scale and of each domain over time (Table 9).

**TABLE 9:** Reliability coefficients of total scale and each domain of Ch-InMLS scale for diabetes mellitus (n = 553).

Domains	Items	Cronbach’s α coefficient	Spearman–Brown split-half reliability	Test–retest reliability
K	10	0.952	0.925	0.865
A	11	0.947	0.944	0.845
P	7	0.908	0.901	0.987
S	8	0.923	0.917	0.936
Ch-InMLS	36	0.945	0.803	0.944

Note: K is short for “knowledge”, A for “attitude”, P for “practice”, and S for “skill”.

#### 3.4.3 Final scale

The scale of Ch-InMLS for patients with DM comprised 36 items and four domains: knowledge, attitude, practice, and skill (Supplementary Material S12); acceptable validity and good reliability of the scale were established.

## 4 Discussion

To best of our knowledge, this is the first study to report the validity and reliability of an assessment scale to comprehensively evaluate the level of insulin medication literacy for patients with diabetes mellitus (DM) in Zhengzhou. According to its results, the Ch-InMLS demonstrated good validity and reliability, indicating that this scale can be used to assess the insulin literacy of DM patients.

We compared our scale with existing scales from the perspective of scale application. Although there were several existing evaluation tools for insulin, they were primarily directed at evaluating psychological status rather than a comprehensive ability to safely and effectively use insulin. For example, the Insulin Treatment Appraisal Scale (ITAS) (Snoek et al., 2007), self-administered Barriers to Insulin Treatment (BIT) questionnaire (Petrak et al., 2007), distress measurement for insulin injections among patients with diabetes (Choi et al., 2023), and the Korean version of Psychological Insulin Resistance (K-PIR) (Song et al., 2016) are designed to evaluate negative attitude toward insulin treatment. In addition, there were also several scales aiming to assess both positive and negative attitudes, such as the Chinese Attitudes to Starting Insulin Questionnaire (Ch-ASIQ) (Fu et al., 2013), Insulin Pump Attitudes Questionnaire (IPA-Questionnaire) (Bergis et al., 2021), Decisional Balance for Insulin Injection (DBII) scale (Hsu et al., 2019), Insulin Treatment Experience Questionnaire (Moock et al., 2010), Insulin Treatment Satisfaction questionnaire (Anderson et al., 2004), Assessing Barriers and Adherence to Insulin Injection technique (Ehrmann et al., 2024), and patient satisfaction with insulin therapy (Cappelleri et al., 2000), which still emphasize subjective cognition and could not objectively evaluate attitude and ability to take insulin. Furthermore, there were several scales designed to evaluate self-management of or self-efficacy about insulin, such as the Insulin Treatment Self-Management Scale (Karahan Okuroglu et al., 2020), Turkish Insulin Treatment Self-management Scale (Çövener Özçelik et al., 2019), and Insulin Therapy Self-efficacy Scale (ITSS) (Nakaue et al., 2019), which are still insufficient to explain the implications of insulin medication literacy, since this includes not only attitude to insulin, but also comprehending insulin related knowledge, practice of routine insulin therapy in daily life, and the abilities and skills to use insulin effectively.

Moreover, we compared our scale with existing scales from the methodology and the results. We developed an insulin medication literacy scale (Ch-InMLS) containing the four domains of knowledge, attitude, practice, skill, based on the conceptual knowledge–attitude–practice model (KAP) framework with “skills” added. A strict, step-by-step approach consisting of item generation, semi-structured interviews, cognitive interviews, two rounds of expert consultation, item selection, construct validity, and reliability analysis was carried out. Petrak et al. (2007) only verified construct validity by EFA and CFA and verified reliability by internal consistency reliability. They lacked systematic research for scale development and pilot survey, which was a weakness of their study. Snoek et al. (2007) simply referred to two studies in the development of their scale, which was inadequate, and the two-factor solution explained only 45% of the total variance. Moreover, item-total correlations (0.34–0.53) for the positive appraisal domain were relatively low. In Karahan Okuroglu et al. (2020), before formal investigation, only face validity was performed with 15 individuals, and they had no pilot survey. Participants were recruited from only one hospital, all of which made the study less convincing.

In content validity analysis, some items that differed from the results of expert consultation were deleted or adjusted so that the scale could accurately and comprehensively reflect the concept under study. For instance, in the first round of Delphi, the item “The use of insulin is the natural progression of diabetes and does not imply the aggravation of the disease” was deleted because it repeated the item “The initiation of insulin therapy is determined by a combination of factors such as my blood sugar level, pancreatic function, complications, and other factors, and does not represent the severity of the disease”, and the expression of former item was less professional than the latter. The item “Injecting insulin indicates failure of pre-insulin treatment” was deleted since the experts mentioned that patients receiving insulin treatment under special conditions could still return to non-insulin therapies. The item “Insulin can prevent damage to liver and kidney function” was deleted because this item seemed too “professional” for patients. the item “Injecting insulin is painful” was deleted because of the possibility of negative guidance. The item “Before injecting medium acting insulin and premixed insulin, I will mix them well” was deleted since there is no need to mix long-acting insulin. The item “Insulin is a physiological hormone secreted by the body that directly lowers blood sugar” was adjusted to the more accurate “Insulin is the only physiological hormone secreted by the body that directly lowers blood sugar.” The item “I know that different insulin cannot be freely converted between them” was adjusted to “I know that there is no arbitrary conversion between medium/long-acting insulin and short/rapid acting insulin” to make it sound more scientific. In the second round of Delphi, the item “Insulin can improve pancreatic function” was deleted since it seemed a little esoteric for patients. The item “Insulin means I have to give up activities I enjoy” was deleted since it is well known that proper exercise is beneficial to disease. The item “The initiation of insulin therapy is determined by a combination of factors such as my blood sugar level, pancreatic function, complications, and other factors and does not represent the severity of the disease” was adjusted to the more accurate “The initiation of insulin therapy is determined by a combination of factors such as my blood sugar level, pancreatic function, complications, and other factors, and does not fully represent the severity of the disease.” The item “I believe that the insulin prescribed by the doctor can prevent or delay the occurrence of complications, such as diabetic kidney disease and diabetic eye disease, etc.” was adjusted to the more accurate “I believe that the insulin prescribed by the doctor can help me control my blood sugar, so as to prevent or delay the occurrence of complications, such as diabetic kidney disease and diabetic eye disease, etc.“

In the pilot survey, the item discrimination analysis, correlation coefficient method, and homogeneity test simultaneously indicated that most items had high relevance and could effectively measure the intended research variables. One exception is the item “Insulin will make others perceive greater sickness”, for which the difference between the high and low score group factors was not significant, Pearson’s correlation coefficients were smaller than the acceptable threshold, and Cronbach’s α coefficient increased after deletion, suggesting that this item had no practical significance; this item was removed. Except for statistical reasons, it could be interpreted as two factors. First, insulin was initially developed by foreigners a century ago and was then introduced to China, which had very scarce in healthcare settings. Thus, people thought that insulin meant greater sickness. But more recently, insulin has been readily available on the market owing to the policy of centralized drug procurement. Second, first- and second-generation insulins were chemically unstable, and side effects were serious; thus, the safe use of insulin was strongly emphasized, deepening the perception that insulin meant greater sickness. Nowadays, with the continuous progress of technology, third-generation insulin has become increasingly stable and safe. Given this background, the item “Insulin will make others perceive greater sickness” was deleted before formal investigation.

Additionally, in the EFA of the present study, the results showed that the KMO test (KMO = 0.944) and Bartlett’s test of sphericity were preferable for factor analysis.

The first domain was “knowledge,” which was consistent with the first step of successful diabetes self-management being patients’ knowledge about the disease (Jiang et al., 2023) and that a reason for low adherence to insulin therapy is poor knowledge of DM and insulin self-injection (Mariye et al., 2019). The knowledge domain accounted for 19.427% of the total variance and it consisted of ten items related to the recognition of DM (item K1), the role of insulin in the treatment of DM (K2, K3, K4, K5), basic information about the insulin being used (K6, K7, K10), and storage conditions for opened and unopened insulin (K8, K9). As is well known, insufficient understanding of the role and status of insulin in the treatment of diabetes is universal. Examples are the belief that glucose can be well controlled just by taking oral medications and insulin, that therapy represented aggravation in disease, and that insulin was dependent or addictive (Wen, et al., 2024; Hussein, et al., 2019). Therefore, an understanding of related knowledge about insulin is essential for establishing insulin medication literacy.

The second domain was “attitude,” accounting for 20.893% of the total variance and was the most influential component of the scale. This domain consisted of 11 items related to personal beliefs about the benefit of insulin (item A1), psychological resistance to insulin (A2, A3), adherence to insulin (A4, A5, A6, A7, A8), the relationship of insulin to diet and exercise (A10), internet insulin literacy (A9), and the pain of insulin injection (A11). “Attitude” meant favorable or unfavorable feelings toward performing insulin management and served as a significant mediator between knowledge and behavior. Several studies have demonstrated that, even with related knowledge, some patients still interrupt or reduce the dose of insulin at will, and some miss their insulin when they go out because of embarrassment and shame (Ellis et al., 2018). Furthermore, there are also patients who are more likely to hold misconceptions about insulin (de Lusignan et al., 2022), and some exaggerate the pain of insulin injections (Liu et al., 2022). All of the above perspectives have a negative effect on acceptance and adherence to insulin therapy. Hence, attitude to insulin treatment was also a crucial indicator for assessing insulin medication literacy.

The third domain was “practice,” accounting for 12.537% of the total variance; it consisted of seven items related to daily behavior under insulin treatment: blood sugar monitoring (P2), dose adjusting (P3, P7), and dealing with special circumstances (P1, P4, P5, P6). In the international consensus about medication literacy, one of the clusters identified was the outcomes and goals of medication literacy (Pouliot, et al., 2018). According to the characteristics of DM, optimal glucose control during insulin use was expected to be achieved. Several studies had demonstrated that self-management, especially the practice of insulin treatment in daily life, improved glycemic control for people with DM (Liang, et al., 2023; Fabrizi et al., 2020). Consequently, practice related to adherence to insulin treatment, the individual management of insulin dosage, communication with medical staff, and self-monitoring of blood glucose behavior was incorporated into the development of insulin medication literacy.

The fourth domain was “skill,” accounting for 14.699% of the total variance; it consisted of eight items related to the injection technique (S1, S3, S5, S6, S7, S8), injection time (S4), and the treatment of hypoglycemia (S2). Unlike injections for in-patient use administered by medical staff, insulin was a special dosage form that mostly needs to be injected by patient themselves. The abilities and skills to use insulin effectively and safely according to printed information and education by medical staff was one of the elements we considered in operationalizing the concept of insulin medication literacy for DM; specifically, skills such as the technique of injection, coping with side effects, and administration time were incorporated into the construction of insulin medication literacy.

CFA was conducted to test goodness-of-fit for the identified four-domain model; IFI was >0.9, the χ2/df value was less <3, RMSEA was <0.08, and PGFI and PNFI were >0.5, both meeting the thresholds recommended. AGFI and GFI were slightly <0.9, RMR was slightly >0.05 and did not exactly meet the thresholds recommended. These indices, however, are likely to be underestimated when the sample size is <300 (Kai, et al., 2018). Additionally, convergent validity was identified by calculating AVE. The results demonstrated that the AVE of the four domains was >0.50, indicating that the variance between domains and associated items exceeded that caused by measurement errors (Acosta-Prado et al., 2020), establishing acceptable convergent validity. Discriminant validity was also assessed by calculating the average variance and squared correlation coefficient among domains. The results demonstrated that the square root of AVE was greater than all possible two-factor correlation coefficients, illustrating that acceptable discriminant validity was established. Overall, the CFA model fit indices of the scale were acceptable, although less than perfect, indicating coherence between the information and the theoretical structure.

Furthermore, later internal consistency evaluations showed that Cronbach’s α exceeded 0.9 for the total scale and each of the domains, indicating that the scale had high internal consistency and that no additional adjustments to the Ch-InMLS were required. The retest reliability coefficient of the scale was 0.944, indicating that the measurement time had little influence on the reliability of this scale and that it had strong time flexibility and stability in assessing Ch-InMLS. Our findings indicated that the scale had good overall reliability, indicating that the scale could adequately measure the level of insulin medication literacy.

## 5 Limitations

Several limitations to the study should be noted. First, owing to constraints of time and resources, participants were recruited from one city (Zhengzhou) by a convenience sampling approach, which was not representative of the entire population. Moreover, we did not assess the participants’ cultural background, which may affect aspects of insulin medication literacy. Second, the investigation of participants was conducted online, so bias in ability to understand may occur in the self-reporting of participants. As with all scales, there was also a risk of overestimating insulin medication literacy due to the shame and fear of low literacy in the current medical culture. Furthermore, we did not evaluate criterion validity due to the lack of a golden criterion to assess the insulin medication literacy of patients with DM.

## 6 Conclusion

Generally, the Ch-InMLS can be applied as a valid and reliable instrument to measure insulin medication literacy amongst patients with diabetes mellitus (DM). It provides policymakers and hospital administrators with an applicable and reliable tool for formulating policies regarding insulin education and training programs. It could be used by medical staff to simplify the insulin regimen, which could in turn improve insulin compliance and improve the health outcomes of patients with DM. In addition, the scale could be integrated into clinical practice as part of routine clinical assessment so that physicians can propose specific interventions without delay when they detect low medication literacy about insulin.

## Data Availability

The original contributions presented in the study are included in the article/Supplementary Material; further inquiries can be directed to the corresponding author.

## References

[B1] Acosta-Prado J. C. López-Montoya O. H. Sanchís-Pedregosa C. Zárate-Torres R. A. (2020). Human resource management and innovative performance in non-profit hospitals: the mediating effect of organizational culture. Front. Psychol. 11, 1422. 10.3389/fpsyg.2020.01422 32636791 PMC7318991

[B2] Anderson R. T. Skovlund S. E. Marrero D. Levine D. W. Meadows K. Brod M. (2004). Development and validation of the insulin treatment satisfaction questionnaire. Clin. Ther. 26 (4), 565–578. 10.1016/s0149-2918(04)90059-8 15189754

[B3] Bergis D. Roos T. Ehrmann D. Schmitt A. Schipfer M. Haak T. (2021). Perceived benefits and barriers regarding CSII treatment: development and psychometric evaluation of the insulin Pump attitudes questionnaire (IPA-Questionnaire). Exp. Clin. Endocrinol. Diabetes 129 (8), 566–573. 10.1055/a-0899-4980 31426110

[B4] Bermeo-Cabrera J. Almeda-Valdes P. Riofrios-Palacios J. Aguilar-Salinas C. A. Mehta R. (2018). Insulin adherence in type 2 diabetes in Mexico: behaviors and barriers. J. Diabetes Res. 2018, 3190849. 10.1155/2018/3190849 30116737 PMC6079463

[B5] Boonpattharatthiti K. Saensook T. Neelapaijit N. Sakunrag I. Krass I. Dhippayom T. (2024). The prevalence of adherence to insulin therapy in patients with diabetes: a systematic review and meta-analysis. Res. Soc. Adm. Pharm. 20 (3), 255–295. 10.1016/j.sapharm.2023.11.009 38104019

[B6] Cappelleri J. C. Gerber R. A. Kourides I. A. Gelfand R. A. (2000). Development and factor analysis of a questionnaire to measure patient satisfaction with injected and inhaled insulin for type 1 diabetes. Diabetes Care 23 (12), 1799–1803. 10.2337/diacare.23.12.1799 11128356

[B7] Chen J. Song Y. Ou L. Wang X. Wang Y. Chen Y. (2023). Development and psychometric evaluation of a self-management behaviours scale in rheumatoid arthritis patients (RA-SMBS). Bmc. Nurs. 22 (1), 40. 10.1186/s12912-023-01173-4 36782215 PMC9926751

[B8] Chen Z. Gu B. X. Tang Y. F. Yan Z. Y. Ni F. D. Cui N. H. (2022). Constructions of the scale of difficulty in the extraction of impacted mandibular third molars by using Delphi method. Beijing Da Xue Xue Bao Yi Xue Ban. 54 (1), 100–104. 10.19723/j.issn.1671-167X.2022.01.016 35165475 PMC8860643

[B9] Choi E. Kim M. S. Cho J. Kim S. Kwon E. K. Kim Y. (2023). Development and validation of a distress measurement for insulin injections among patients with diabetes. Sci. Rep. 13 (1), 11725. 10.1038/s41598-023-38982-1 37474582 PMC10359257

[B10] Cleland J. (1973). A critique of KAP studies and some suggestions for their improvement. Stud. Fam. Plan. 4 (2), 42–47. 10.2307/1964829 4691025

[B11] Cloete L. (2022). Diabetes mellitus: an overview of the types, symptoms, complications and management. Nurs. Stand. 37 (1), 61–66. 10.7748/ns.2021.e11709 34708622

[B12] Çövener Özçelik Ç. Aktaş E. Şen Celasin N. Karahan Okuroğlu G. Şahin Ş. (2019). The development and validation of a Turkish insulin treatment self-management scale child form (ages 8-18) and parent form. J. Clin. Res. Pediatr. Endocrinol. 11 (3), 278–286. 10.4274/jcrpe.galenos.2019.2019.0026 30905141 PMC6745460

[B13] Dabas H. Sarin J. Madhu S. V. (2023). Insulin adherence in adolescents with type 1 diabetes mellitus. Indian. J. Endocrinol. Metab. 27 (5), 394–397. 10.4103/ijem.ijem_294_22 38107739 PMC10723607

[B14] de Lusignan S. McGovern A. Hinton W. Whyte M. Munro N. Williams E. D. (2022). Barriers and facilitators to the initiation of injectable therapies for type 2 diabetes mellitus: a mixed methods study. Diabetes Ther. Res. Treat. Educ. Diabetes Relat. Disord. 13 (10), 1789–1809. 10.1007/s13300-022-01306-z PMC9500132 36050586

[B15] Ehrmann D. Kulzer B. Wienbarg I. Sieber J. Weber S. Haak T. (2024). Assessing barriers and adherence to insulin injection technique in people with diabetes: development and validation of new assessment tools. J. Diabetes Sci. Technol. 18 (6), 1362–1369. 10.1177/19322968231175920 37209023 PMC11529062

[B16] Ellis K. Mulnier H. Forbes A. (2018). Perceptions of insulin use in type 2 diabetes in primary care: a thematic synthesis. BMC Fam. Pract. 19 (1), 70. 10.1186/s12875-018-0753-2 29788908 PMC5964885

[B17] Elo S. Kyngäs H. (2008). The qualitative content analysis process. J. Adv. Nurs. 62 (1), 107–115. 10.1111/j.1365-2648.2007.04569.x 18352969

[B18] Emmerton L. M. Mampallil L. Kairuz T. McKauge L. M. Bush R. A. (2012). Exploring health literacy competencies in community pharmacy. Health Expect. 15 (1), 12–22. 10.1111/j.1369-7625.2010.00649.x 21122042 PMC5060605

[B19] Fabrizi D. Rebora P. Luciani M. Di Mauro S. Valsecchi M. G. Ausili D. (2020). How do self-care maintenance, self-care monitoring, and self-care management affect glycated haemoglobin in adults with type 2 diabetes? A multicentre observational study. Endocrine 69 (3), 542–552. 10.1007/s12020-020-02354-w 32504379

[B20] Farsaei S. Radfar M. Heydari Z. Abbasi F. Qorbani M. (2014). Insulin adherence in patients with diabetes: risk factors for injection omission. Prim. Care. Diabetes 8 (4), 338–345. 10.1016/j.pcd.2014.03.001 24721139

[B21] Fu S. N. Chin W. Y. Wong C. K. Yeung V. T. Yiu M. P. Tsui H. Y. (2013). Development and validation of the Chinese Attitudes to Starting Insulin Questionnaire (Ch-ASIQ) for primary care patients with type 2 diabetes. PLoS. One. 8 (11), e78933. 10.1371/journal.pone.0078933 24236071 PMC3827341

[B22] Gnägi R. Zúñiga F. Brunkert T. Meyer-Massetti C. (2022). Development of a medication literacy assessment instrument (MELIA) for older people receiving home care. J. Adv. Nurs. 78 (12), 4210–4220. 10.1111/jan.15429 36052608 PMC9826207

[B23] Hair J. F. Jr. Black W. C. Babin B. J. (2009). Multivariate Data Analysis. Editor Anderson R. E., 7th Edn. (New York, NY: Prentice Hall).

[B25] Hsu H. C. Chen S. Y. Huang Y. C. Wang R. H. Lee Y. J. An L. W. (2019). Decisional balance for insulin injection: scale development and psychometric testing. J. Nurs. Res. JNR 27 (5), e42. 10.1097/jnr.0000000000000316 30807441 PMC6752819

[B26] Hu L. T. Bentler P. M. (1998). Fit indices in covariance structure modeling: sensitivity to underparameterized model misspecification. Psychol. Methods 3 (4), 424–453. 10.1037/1082-989X.3.4.424

[B27] Hussein A. Mostafa A. Areej A. Mona A. M. Shimaa A. Najd A. G. (2019). The perceived barriers to insulin therapy among type 2 diabetic patients. Afr. health Sci. 19 (1), 1638–1646. 10.4314/ahs.v19i1.39 31148993 PMC6531943

[B28] Jang S. M. Jiang R. Grabe D. Pai A. B. (2019). Assessment of literacy and numeracy skills related to non-steroidal anti-inflammatory drug labels. SAGE. Open. Med. 7, 2050312119834119. 10.1177/2050312119834119 30873281 PMC6407168

[B29] Jiang T. Li A. Zhang M. Zhou Z. Wang L. Zhang X. (2023). Measuring self-management among people with diabetes mellitus: a systematic review of patient-reported diabetes-specific instruments in English and Chinese. Adv. Ther. 40 (3), 769–813. 10.1007/s12325-022-02361-5 36607543

[B30] Jünger S. Payne S. A. Brine J. Radbruch L. Brearley S. G. (2017). Guidance on Conducting and REporting DElphi Studies (CREDES) in palliative care: recommendations based on a methodological systematic review. Palliat. Med. 31 (8), 684–706. 10.1177/0269216317690685 28190381

[B33] Kai W. Fangyao C. Ming T. Pingyan C. (2018). A New Corrected-Good-of-Fit Index (CGFI) for Model Evaluation in Structural Equation Modeling. Chinese Journal of Health Statistics. 35 (3), 349–354. 10.3969/j.issn.1002-3674.2018.03.006

[B31] Karahan Okuroglu G. Karaçanta Atbaş S. Ecevit Alpar Ş. (2020). Development, reliability, and validity of the insulin treatment self-management scale. Nurs. Pract. 26 (5), e12814. 10.1111/ijn.12814 31880038

[B32] Kelley T. L. (1939). The selection of upper and lower groups for the validation of test Items. J. Educ. Psychol. 30, 17–24. 10.1037/h0057123

[B34] Koo T. K. Li M. Y. (2016). A guideline of selecting and reporting intraclass correlation coefficients for reliability research. Chiropr. Med. 15 (2), 155–163. 10.1016/j.jcm.2016.02.012 PMC4913118 27330520

[B35] Lee S. H. Yoon K. H. (2021). A century of progress in diabetes care with insulin: a history of innovations and foundation for the future. Metab. J. 45 (5), 629–640. 10.4093/dmj.2021.0163 PMC8497924 34610718

[B36] Liang W. Lo S. H. S. Chow K. M. Zhong J. Ni X. (2023). Perception of self-management and glycaemic control in people with type 2 diabetes receiving insulin injection therapy: a qualitative study. Prim. care diabetes 17 (6), 587–594. 10.1016/j.pcd.2023.08.006 37658019

[B37] Liu C. De Roza J. Ooi C. W. Mathew B. K. Tang W. E. (2022). Impact of patients’ beliefs about insulin on acceptance and adherence to insulin therapy: a qualitative study in primary care. BMC Prim. care 23 (1), 15. 10.1186/s12875-022-01627-9 35172774 PMC8776322

[B38] Liu Z. H. Liu P. (2010). PASW/SPSS Statistics Chinese Edition Statistical Analysis Course. 3rd edn. Beijing: Publishing House of Electronics Industry.

[B39] Lovic D. Piperidou A. Zografou I. Grassos H. Pittaras A. Manolis A. (2020). The growing epidemic of diabetes mellitus. Vasc. Pharmacol. 18 (2), 104–109. 10.2174/1570161117666190405165911 30961501

[B40] Lynn M. R. (1986). Determination and quantification of content validity. Nurs. Res. 35 (6), 382–386. 10.1097/00006199-198611000-00017 3640358

[B41] Ma A. J. Dong J. Wei Y. Q. Fang K. Xie C. Jiang B. (2020). Comprehensive control rate and related factros of diabetes mellitus in Beijing. Prev. Med. 54 (11), 1283–1288. 10.3760/cma.j.cn112150-20200616-00887 33147930

[B42] Mariye T. Girmay A. Birhanu T. Tasew H. Teklay G. Baraki Z. (2019). Adherence to insulin therapy and associated factors among patients with diabetes mellitus in public hospitals of Central Zone of Tigray, Ethiopia, 2018: a cross-sectional study. Pan. Afr. Med. J. 33, 309. 10.11604/pamj.2019.33.309.17547 31692777 PMC6815482

[B43] McPherson S. Reese C. Wendler M. C. (2018). Methodology update: Delphi studies. Nurs. Res. 67 (5), 404–410. 10.1097/NNR.0000000000000297 30052591

[B44] Metsmuuronen J. (2020). Generalized discrimination index. Int. J. Educ. Method 6, 237–257. 10.12973/ijem.6.2.237

[B45] Moock J. Hessel F. Ziegeler D. Kubiak T. Kohlmann T. (2010). Development and testing of the insulin treatment experience questionnaire (ITEQ). patient 3 (1), 45–58. 10.2165/11319510-000000000-00000 22273275

[B133] Mulaik S. A. James L. R. Van Alstine J. Bennet N. Lind S. Stilwell C. D. (1989). Evaluation of goodness-of-fit indices for structural equation models. Psychol. Bull. 105 430–455. 10.1037/0033-2909.105.3.430

[B46] Nakaue J. Koizumi M. Nakajima H. Okada S. Mohri T. Akai Y. (2019). Development of a self-efficacy questionnaire, 'insulin therapy self-efficacy scale (ITSS)', for insulin users in Japanese: the self-efficacy-Q study. J. diabetes investigation 10 (2), 358–366. 10.1111/jdi.12914 PMC6400175 30136385

[B47] Neiva Pantuzza L. L. Nascimento E. D. Crepalde-Ribeiro K. Botelho S. F. Parreiras Martins M. A. Camila de Souza Groia Veloso R. (2022). Medication literacy: a conceptual model. Res. Soc. Adm. Pharm. 18 (4), 2675–2682. 10.1016/j.sapharm.2021.06.003 34134939

[B48] Pantuzza L. L. N. do Nascimento E. Botelho S. F. da Rocha A. L. P. Martins M. A. P. do Nascimento M. M. G. (2023). Development and content validation of the medication literacy test for older adults (TELUMI). Arch. Gerontol. Geriatr. 112, 105027. 10.1016/j.archger.2023.105027 37080136

[B49] Petrak F. Stridde E. Leverkus F. Crispin A. A. Forst T. Pfützner A. (2007). Development and validation of a new measure to evaluate psychological resistance to insulin treatment. Diabetes Care 30 (9), 2199–2204. 10.2337/dc06-2042 17575092

[B50] Pett A. M. Lackey N. R. Sullivan J. J. (2003). “Making sense of factor analysis: the use of factor analysis for instrument development,” in Health Care Research (Thousand Oaks, CA: Sage). 10.4135/9781412984898

[B51] Peyrot M. Barnett A. H. Meneghini L. F. Schumm-Draeger P. M. (2012). Insulin adherence behaviours and barriers in the multinational global attitudes of patients and physicians in insulin therapy study. Diabet. Med. 29 (5), 682–689. 10.1111/j.1464-5491.2012.03605.x 22313123 PMC3433794

[B52] Polit D. F. Beck C. T. Owen S. V. (2007). Is the CVI an acceptable indicator of content validity? Appraisal and recommendations. Res. Nurs. Health. 30 (4), 459–467. 10.1002/nur.20199 17654487

[B53] Pouliot A. Vaillancourt R. Stacey D. Suter P. (2018). Defining and identifying concepts of medication literacy: an international perspective. Res. Soc. Adm. Pharm. 14 (9), 797–804. 10.1016/j.sapharm.2017.11.005 29191647

[B54] Riddle M. C. (2021). The current schemes of insulin therapy: pro and contra. Diabetes. Res. Clin. Pract. 175, 108817. 10.1016/j.diabres.2021.108817 33865916

[B55] Sauceda J. A. Loya A. M. Sias J. J. Taylor T. Wiebe J. S. Rivera J. O. (2012). Medication literacy in Spanish and English: psychometric evaluation of a new assessment tool. J. Am. Pharm. Assoc. 52 (6), e231–e240. 10.1331/JAPhA.2012.11264 23229985

[B56] Shreffler-Grant J. Weinert C. Nichols E. (2014). Instrument to measure health literacy about complementary and alternative medicine. J. Nurs. Meas. 22 (3), 489–499. 10.1891/1061-3749.22.3.489 25608434 PMC5185466

[B57] Skriver L. K. L. Nielsen M. W. Walther S. Nørlev J. D. Hangaard S. (2023). Factors associated with adherence or nonadherence to insulin therapy among adults with type 2 diabetes mellitus: a scoping review. J. Diabetes. Complicat. 37 (10), 108596. 10.1016/j.jdiacomp.2023.108596 37651772

[B58] Snoek F. J. Skovlund S. E. Pouwer F. (2007). Development and validation of the insulin treatment appraisal scale (ITAS) in patients with type 2 diabetes. Health Qual. Life Outcomes 5, 69. 10.1186/1477-7525-5-69 18096074 PMC2241589

[B59] Song Y. Jeon Y. Cho J. Kim B. (2016). Development of a psychological insulin resistance scale for Korean patients with diabetes. J. Korean Acad. Nurs. 46 (6), 813–823. 10.4040/jkan.2016.46.6.813 28077829

[B60] Stilley C. S. Terhorst L. Flynn W. B. Fiore R. M. Stimer E. D. (2014). Medication health literacy measure: development and psychometric properties. Nurs. Meas. 22 (2), 213–222. 10.1891/1061-3749.22.2.213 PMC4580338 25255674

[B61] Sun J. (2005). Assessing goodness of fit in confirmatory factor analysis. Eval. Couns. Dev. 37, 240–256. 10.1080/07481756.2005.11909764

[B63] Ubavić S. Bogavac-Stanojević N. Jović-Vraneš A. Krajnović D. (2018). Understanding of information about medicines use among parents of pre-school children in Serbia: parental pharmacotherapy literacy questionnaire (PTHL-SR). Int. J. Environ. Res. Public Health 15 (5), 977. 10.3390/ijerph15050977 29757928 PMC5982016

[B64] Vervloet M. van Dijk L. Rademakers J. J. D. J. M. Bouvy M. L. De Smet P. A. G. M. Philbert D. (2018). Recognizing and addressing limited PHarmaceutical literacy: development of the RALPH interview guide. Res. Soc. Adm. Pharm. 14 (9), 805–811. 10.1016/j.sapharm.2018.04.031 29724680

[B65] Wang Z. Wu Y. Wu J. Wang M. Wang X. Wang J. (2021). Trends in prevalence and incidence of type 2 diabetes among adults in Beijing, China, from 2008 to 2017. Diabet. Med. 38 (9), e14487. 10.1111/dme.14487 33278034

[B66] Wen S. Ruan Y. Shi Z. Dan S. Zhou L. (2024). The barriers to insulin therapy initiation in type 2 diabetes patients: a study of general practitioner perceptions in huinan community in south shanghai. Diabetes, metabolic syndrome Obes. targets Ther. 17, 393–405. 10.2147/DMSO.S446349 PMC10822111 38283634

[B67] Wu M. L. (2010). Statistical Analysis Practices in Questionnaire Development. Chongqing: Chongqing University Press.

[B68] Xu Y. Wang L. He J. Bi Y. Li M. Wang T. (2013). Prevalence and control of diabetes in Chinese adults. JAMA 310 (9), 948–959. 10.1001/jama.2013.168118 24002281

[B69] Yavuz D. G. Ozcan S. Deyneli O. (2015). Adherence to insulin treatment in insulin-naïve type 2 diabetic patients initiated on different insulin regimens. Patient Prefer Adherence 9, 1225–1231. 10.2147/PPA.S87935 26346988 PMC4556254

[B70] Yeh Y. C. Lin H. W. Chang E. H. Huang Y. M. Chen Y. C. Wang C. Y. (2017). Development and validation of a Chinese medication literacy measure. Health Expect. 20 (6), 1296–1301. 10.1111/hex.12569 28474423 PMC5689244

[B71] Zhang N. Wang L. Ouyang Y. Q. Redding S. (2021). Survey on medication information literacy and influencing factors among pregnant Chinese women. J. Matern. Fetal Neonatal. Med. 34 (10), 1619–1626. 10.1080/14767058.2019.1642869 31331258

[B72] Zhong Z. Ma G. Zheng F. Duan Y. Ding S. Luo A. (2020a). Medication literacy in a cohort of Chinese patients discharged with essential hypertension. Front. Public Health 7, 385. 10.3389/fpubh.2019.00385 31998676 PMC6962135

[B73] Zhong Z. Shi S. Duan Y. Shen Z. Zheng F. Ding S. (2020b). The development and psychometric assessment of Chinese medication literacy scale for hypertensive patients (C-MLSHP). Front. Pharmacol. 11, 490. 10.3389/fphar.2020.00490 32425773 PMC7203424

