# Supplementary material for: Development and validation of a Chinese insulin medication literacy scale for patients with diabetes mellitus
Source: Front Pharmacol. 2025 Apr 2;16:1477050. doi: 10.3389/fphar.2025.1477050 (PMC11999841; doi:10.3389/fphar.2025.1477050)
Supplement: Supplementary file 1 [file Supplementaryfile6.docx]

Supplementary file 6

Pearson’s correlation coefficients between item A1 to A12 and total score of Attitude domain

|  | *P* | *sig(bilateral)* |
| --- | --- | --- |
| A1 | 0.765 | 0.000 |
| A2 | 0.914 | 0.000 |
| A3 | 0.846 | 0.000 |
| A4 | 0.828 | 0.000 |
| A5 | 0.734 | 0.000 |
| A6 | 0.823 | 0.000 |
| A7 | 0.748 | 0.000 |
| A8 | 0.821 | 0.000 |
| A9 | 0.835 | 0.000 |
| A10 | 0.738 | 0.000 |
| A11 | 0.790 | 0.000 |
| A12 | **-0.064** | 0.000 |

Note: A is short for attitude.
