# Supplementary material for: Development and validation of a Chinese insulin medication literacy scale for patients with diabetes mellitus
Source: Front Pharmacol. 2025 Apr 2;16:1477050. doi: 10.3389/fphar.2025.1477050 (PMC11999841; doi:10.3389/fphar.2025.1477050)
Supplement: Supplementary file 2 [file Supplementaryfile10.docx]

Supplementary file 10

Cronbach' s α coefficient after item deletion for homogeneity test

|  | Cronbach' s α for the scale | Cronbach' s α coefficient after item deletion |
| --- | --- | --- |
|  | 0.928 |  |
| K1 |  | 0.924 |
| K2 |  | 0.924 |
| K3 |  | 0.925 |
| K4 |  | 0.925 |
| K5 |  | 0.924 |
| K6 |  | 0.926 |
| K7 |  | 0.927 |
| K8 |  | 0.927 |
| K9 |  | 0.926 |
| K10 |  | 0.927 |
| A1 |  | 0.926 |
| A2 |  | 0.924 |
| A3 |  | 0.925 |
| A4 |  | 0.925 |
| A5 |  | 0.927 |
| A6 |  | 0.925 |
| A7 |  | 0.925 |
| A8 |  | 0.925 |
| A9 |  | 0.925 |
| A10 |  | 0.928 |
| A11 |  | 0.924 |
| A12 |  | **0.935** |
| P1 |  | 0.927 |
| P2 |  | 0.926 |
| P3 |  | 0.926 |
| P4 |  | 0.924 |
| P5 |  | 0.926 |
| P6 |  | 0.925 |
| P7 |  | 0.925 |
| S1 |  | 0.926 |
| S2 |  | 0.926 |
| S3 |  | 0.925 |
| S4 |  | 0.926 |
| S5 |  | 0.926 |
| S6 |  | 0.927 |
| S7 |  | 0.926 |
| S8 |  | 0.926 |

Note: K is short for knowledge; A is short for attitude; P is short for practice; S is short for skill.
