# Supplementary material for: Development and validation of a Chinese insulin medication literacy scale for patients with diabetes mellitus
Source: Front Pharmacol. 2025 Apr 2;16:1477050. doi: 10.3389/fphar.2025.1477050 (PMC11999841; doi:10.3389/fphar.2025.1477050)
Supplement: Supplementary file 3 [file Supplementaryfile1.docx]

Supplementary file 1

**Guidelines for Reporting Reliability and Agreement Studies (GRRAS).**

TITLE AND ABSTRACT

Item 1: Identify in title or abstract that interrater/intrarater reliability or agreement was investigated.

In the abstract, internal consistency reliability which was the same as interrater reliability was investigated.

INTRODUCTION

Item 2: Name and describe the diagnostic or measurement device of interest explicitly.

The measurement device of interest was explicitly named and described.

Item 3: Specify the subject population of interest

The subject population of interest was specified.

Item 4: Specify the rater population of interest (if applicable).

The rater population of interest was specified.

Item 5: Describe what is already known about reliability and agreement and provide a rationale for the study (if applicable).

What is already known about reliability and agreement and a rationale for the study had been describe.

Methods

Item 6: Explain how the sample size was chosen. State the determined number of raters, subjects/objects, and replicate observations.

The above was explained.

Item 7: Describe the sampling method

Sampling method was described.

Item 8: Describe the measurement/rating process (e.g., time interval between repeated measurements, availability of clinical information, blinding).

The above was explained.

Item 9: State whether measurements/ratings were conducted independently.

Measurements/ratings were conducted independently as was stated in the paper.

Item 10: Describe the statistical analysis.

Statistical analysis was described in the paper.

Results

Item 11: State the actual number of raters and subjects/objects that were included and the number of replicate observations that were conducted.

The actual number of raters and subjects/objects that were included and the number of replicate observations that were conducted was stated in the paper.

Item 12: Describe the sample characteristics of raters and subjects (e.g., training, experience).

The sample characteristics of raters and subjects were described.

Item 13: Report estimates of reliability and agreement, including measures of statistical uncertainty.

Estimates of reliability and agreement was reported.

Discussion

Item 14: Discuss the practical relevance of results.

The above was explained.

Item 15: Provide detailed results if possible (e.g., online).

Detailed results were provided.

**The Quality Appraisal of Reliability Studies (QAREL) checklist**

**Item 1 Was the test evaluated in a sample of subjects who were representative of those to whom the authors intended the results to be applied?**

**√YES NO Unclear N/A**

**Item 2 Was the test performed by raters who were representative of those to whom the authors intended the results to be applied?**

**√YES NO Unclear N/A**

**Item 3 Were raters blinded to the findings of other raters during the study?**

**√YES NO Unclear N/A**

**Item 4 Were raters blinded to their own prior findings of the test under evaluation?**

**√YES NO Unclear N/A**

**Item 5 Were raters blinded to the subjects’ disease status or the results of the accepted reference standard for the target disorder (or variable) being evaluated?**

**√YES NO Unclear N/A**

**Item 6 Were raters blinded to clinical information that was not intended to form part of the study design or testing procedure?**

**√YES NO Unclear N/A**

**Item 7 Were raters blinded to additional cues that are not part of the test?**

**√YES NO Unclear N/A**

**Item 8 Was the order of examination varied?**

**YES NO Unclear √N/A**

**Item 9 Was the stability (or theoretical stability) of the variable being measured taken into account when determining the suitability of the time interval among repeated measures?**

**√YES NO Unclear N/A**

**Item 10 Was the test applied correctly and interpreted appropriately?**

**√YES NO Unclear N/A**

**Item 11 Were appropriate statistical measures of agreement used?**

**√YES NO Unclear N/A**
