# Supplementary material for: Development and validation of a Chinese insulin medication literacy scale for patients with diabetes mellitus
Source: Front Pharmacol. 2025 Apr 2;16:1477050. doi: 10.3389/fphar.2025.1477050 (PMC11999841; doi:10.3389/fphar.2025.1477050)
Supplement: Supplementary file 4 [file Supplementaryfile5.docx]

Supplementary file 5

Pearson’s correlation coefficients between item K1 to K10 and total score of Knowledge domain

|  | *P* | *sig(bilateral)* |
| --- | --- | --- |
| K1 | 0.872 | 0.000 |
| K2 | 0.950 | 0.000 |
| K3 | 0.842 | 0.000 |
| K4 | 0.848 | 0.000 |
| K5 | 0.941 | 0.000 |
| K6 | 0.786 | 0.000 |
| K7 | 0.797 | 0.000 |
| K8 | 0.747 | 0.000 |
| K9 | 0.803 | 0.000 |
| K10 | 0.806 | 0.000 |

Note: K is short for knowledge.
