# Supplementary material for: Development and validation of a Chinese insulin medication literacy scale for patients with diabetes mellitus
Source: Front Pharmacol. 2025 Apr 2;16:1477050. doi: 10.3389/fphar.2025.1477050 (PMC11999841; doi:10.3389/fphar.2025.1477050)
Supplement: Supplementary file 5 [file Supplementaryfile11.docx]

Supplementary file 11

Communalities of each item

|  | Extract |
| --- | --- |
| K1 | 0.562^#^ |
| K2 | 0.611^#^ |
| K3 | 0.450^#^ |
| K4 | 0.467^#^ |
| K5 | 0.608^#^ |
| K6 | 0.465^#^ |
| K7 | 0.408^#^ |
| K8 | 0.408^#^ |
| K9 | 0.506^#^ |
| K10 | 0.439^#^ |
| A1 | 0.222^#^ |
| A2 | 0.315^#^ |
| A3 | 0.281^#^ |
| A4 | 0.225^#^ |
| A5 | 0.110 |
| A6 | 0.268^#^ |
| A7 | 0.248^#^ |
| A8 | 0.250^#^ |
| A9 | 0.259^#^ |
| A10 | 0.087 |
| A11 | 0.342^#^ |
| A12 | 0.042 |
| P1 | 0.216^#^ |
| P2 | 0.286^#^ |
| P3 | 0.320^#^ |
| P4 | 0.423^#^ |
| P5 | 0.436^#^ |
| P6 | 0.364^#^ |
| P7 | 0.332^#^ |
| S1 | 0.301^#^ |
| S2 | 0.385^#^ |
| S3 | 0.450^#^ |
| S4 | 0.251^#^ |
| S5 | 0.304^#^ |
| S6 | 0.210^#^ |
| S7 | 0.350^#^ |
| S8 | 0.299^#^ |

Note: K is short for knowledge; A is short for attitude; P is short for practice; S is short for skill.

^#^ communality-value <0.2.
