# Supplementary material for: Development and validation of a Chinese insulin medication literacy scale for patients with diabetes mellitus
Source: Front Pharmacol. 2025 Apr 2;16:1477050. doi: 10.3389/fphar.2025.1477050 (PMC11999841; doi:10.3389/fphar.2025.1477050)
Supplement: Supplementary file 6 [file Supplementaryfile3.docx]

Supplementary file 3

**Content validity**

**The details information** on the number of items and domains revised or deleted from Two rounds of expert consultation.

| **The first round** | |
| --- | --- |
| **Deleted five items** | Item 5: The use of insulin is the natural progression of diabetes and does not imply the aggravation of the disease.  Item 6: Injecting insulin indicates failure of pre-insulin treatment.  Item 7: Insulin can prevent damage to liver and kidney function.  Item 22: Injecting insulin is painful.  Item 39: Before injecting medium acting insulin and premixed insulin, I will mix them well. |
| **Revised two items** | Item 2: “Insulin is a physiological hormone secreted by the body that directly lowers blood sugar” was modified as " Insulin is **the only** physiological hormone secreted by the body that directly lowers blood sugar".  Item 14: “I know that **different insulin cannot be freely converted between them**” was modified as " I know that **there is no arbitrary conversion between medium/long-acting insulin and short/rapid acting insulin**". |
| **Results** | The scale comprised of 39 items and 4 dimensions. |
| **The second round** | |
| **Deleted two items** | Item 8: Insulin can improve pancreatic function.  Item 26: Insulin means I have to give up activities I enjoy. |
| **Revised two items** | Item 4: “The initiation of insulin therapy……, and does not represent the severity of the disease” was modified as “The initiation of insulin therapy……, and does not **fully** represent the severity of the disease”.  Item 15: “I believe that the insulin prescribed by the doctor can prevent or delay……” was modified as “I believe that the insulin prescribed by the doctor can **help me control my blood sugar, so as to prevent or delay** ……”. |
| **Results** | Finally, the scale comprised of 37 items and 4 dimensions. |
