# Supplementary material for: Development and validation of a Chinese insulin medication literacy scale for patients with diabetes mellitus
Source: Front Pharmacol. 2025 Apr 2;16:1477050. doi: 10.3389/fphar.2025.1477050 (PMC11999841; doi:10.3389/fphar.2025.1477050)
Supplement: Supplementary file 7 [file Supplementaryfile9.docx]

Supplementary file 9

Pearson’s correlation coefficients between each item and total score of the scale

|  | *P* | *sig(bilateral)* |
| --- | --- | --- |
| K1 | 0.666 | 0.000 |
| K2 | 0.688 | 0.000 |
| K3 | 0.586 | 0.000 |
| K4 | 0.602 | 0.000 |
| K5 | 0.670 | 0.000 |
| K6 | 0.569 | 0.000 |
| K7 | 0.528 | 0.000 |
| K8 | 0.536 | 0.000 |
| K9 | 0.608 | 0.000 |
| K10 | 0.566 | 0.000 |
| A1 | 0.559 | 0.000 |
| A2 | 0.672 | 0.000 |
| A3 | 0.627 | 0.000 |
| A4 | 0.576 | 0.000 |
| A5 | 0.437 | 0.000 |
| A6 | 0.615 | 0.000 |
| A7 | 0.580 | 0.000 |
| A8 | 0.595 | 0.000 |
| A9 | 0.612 | 0.000 |
| A10 | 0.406 | 0.000 |
| A11 | 0.668 | 0.000 |
| A12 | **0.130** | 0.000 |
| P1 | 0.470 | 0.000 |
| P2 | 0.554 | 0.000 |
| P3 | 0.565 | 0.000 |
| P4 | 0.653 | 0.000 |
| P5 | 0.643 | 0.000 |
| P6 | 0.605 | 0.000 |
