# Supplementary material for: Development and validation of a Chinese insulin medication literacy scale for patients with diabetes mellitus
Source: Front Pharmacol. 2025 Apr 2;16:1477050. doi: 10.3389/fphar.2025.1477050 (PMC11999841; doi:10.3389/fphar.2025.1477050)
Supplement: Supplementary file 8 [file Supplementaryfile7.docx]

Supplementary file 7

Pearson’s correlation coefficients between item P1 to P7 and total score of Practice domain

|  | *P* | *sig(bilateral)* |
| --- | --- | --- |
| P1 | 0.695 | 0.000 |
| P2 | 0.765 | 0.000 |
| P3 | 0.790 | 0.000 |
| P4 | 0.803 | 0.000 |
| P5 | 0.870 | 0.000 |
| P6 | 0.816 | 0.000 |
| P7 | 0.811 | 0.000 |

Note: P is short for practice.
