# Supplementary material for: Development and validation of a Chinese insulin medication literacy scale for patients with diabetes mellitus
Source: Front Pharmacol. 2025 Apr 2;16:1477050. doi: 10.3389/fphar.2025.1477050 (PMC11999841; doi:10.3389/fphar.2025.1477050)
Supplement: Supplementary file 9 [file Supplementaryfile8.docx]

Supplementary file 8

Pearson’s correlation coefficients between item S1 to S8 and total score of Skill domain

|  | *P* | *sig(bilateral)* |
| --- | --- | --- |
| S1 | 0.813 | 0.000 |
| S2 | 0.858 | 0.000 |
| S3 | 0.826 | 0.000 |
| S4 | 0.797 | 0.000 |
| S5 | 0.746 | 0.000 |
| S6 | 0.701 | 0.000 |
| S7 | 0.845 | 0.000 |
| S8 | 0.797 | 0.000 |

Note: S is short for skill.
