# Supplementary material for: Development and validation of a Chinese insulin medication literacy scale for patients with diabetes mellitus
Source: Front Pharmacol. 2025 Apr 2;16:1477050. doi: 10.3389/fphar.2025.1477050 (PMC11999841; doi:10.3389/fphar.2025.1477050)
Supplement: Supplementary file 10 [file Supplementaryfile2.docx]

Supplementary file 2

**Development of Ch-InMLS**

**The details information** on the number of items and domains revised or deleted from **Item generation，Semi-structured interviews, Cognitive interviews.**

| **Item generation** | | |
| --- | --- | --- |
| **40 items** | Knowledge | Item 1: Diabetes is a disease caused by insulin deficiency and/or utilization disorder.  Item 2: Insulin is a physiological hormone secreted by the body that directly lowers blood sugar.  Item 3: Insulin therapy is one of the effective ways to lower blood sugar.  Item 4: The initiation of insulin therapy is determined by a combination of factors such as my blood sugar level, pancreatic function, complications, and other factors, and does not represent the severity of the disease.  Item 5: The use of insulin is the natural progression of diabetes and does not imply the aggravation of the disease.  Item 6: Injecting insulin indicates failure of pre-insulin treatment.  Item 7: Insulin can prevent damage to liver and kidney function.  Item 8: Insulin can improve pancreatic function.  Item 9: Insulin therapy is an exogenous supplement or replacement for insulin deficiency in the body, which does not cause dependence or addiction in the body.  Item 10: I know the name, dosage, and type of insulin I use.  Item 11: I keep an eye on the expiration date of the insulin I use.  Item 12: I know that unopened insulin should be refrigerated at 2-8 °C, avoiding freezing and direct sunlight.  Item 13: I know that insulin that has been opened does not need to be refrigerated at 2-8 ℃, but can be stored at room temperature below 30 ℃ and must be used up within 4 weeks.  Item 14: I know that different insulin cannot be freely converted between them. |
|  | Attitude | Item 15: I believe that the insulin prescribed by the doctor can prevent or delay the occurrence of complications, such as diabetic kidney disease and diabetic eye disease, etc.  Item 16: When using insulin outside, I can effectively avoid or view the potential awkwardness correctly.  Item 17: When I feel good, I can reduce my insulin dose at will.  Item 18: When I feel good, I can stop insulin.  Item 19: When blood sugar control reaches the target value, I can reduce insulin dosage.  Item 20: When blood sugar control reaches the target value, I can stop insulin.  Item 21: I think it is not a big deal to reduce, miss and stop insulin privately.  Item 22: Injecting insulin is painful.  Item 23: Insulin means I have to give up activities I enjoy.  Item 24: When using insulin, there is no need to control diet and exercise.  Item 25: When injecting insulin, there is basically no pain, just like being bitten by a mosquito.  Item 26: Insulin will make others perceive greater sickness. |
|  | Practice | Item 27: I will purchase insulin according to the doctor's prescription.  Item 28: I will monitor my blood sugar under the guidance of my doctor.  Item 29: I will adjust my insulin dose according to my blood sugar level under the guidance of my doctor.  Item 30: When I have questions about insulin, I will consult medical staff (doctors, nurses, pharmacists).  Item 31: When I forget to inject insulin, I know how to deal with it.  Item 32: I will go to the hospital for regular follow-up and adjust the insulin dosage. |
|  | Skill | Item 33: I'll rotate the injection site each time.  Item 34: I know how to deal with hypoglycemic symptoms.  Item 35: I change my insulin needles each time.  Item 36: I will inject insulin at the time recommended by my doctor (such as half an hour before meals, immediately before meals, before bedtime, etc.).  Item 37: I will check the insulin injection site each time, avoiding areas with sunken skin, hard knots, and bruises.  Item 38: I will keep the needle under the skin for at least 10 seconds after insulin injection before pulling it out to prevent drug leakage.  Item 39: Before injecting medium acting insulin and premixed insulin, I will mix them well.  Item 40: I exhaust gas before injecting insulin to ensure accurate dosage. |
| **Semi-structured interviews** | | |
| **Added**  **4 items** |  | 1. A new item "When I inject insulin, I worry that others will know that I have diabetes", was added to the “**Attitude**” dimension.  2. A new item "I will look upon insulin related information on the Internet objectively and rationally", was added to the “**Attitude**” dimension.  3. A new item "During the use of insulin, if I experience any discomfort, I will promptly report it to medical staff", was added to the “**Practice**” dimension.  4. A new item "Before using unopened insulin, I rewarm it before using it", was added to the “**Skill**” dimension. |
| **Results** |  | The scale comprised of 44 items and 4 dimensions. |
| **Cognitive interviews** | | |
| **Revised four items** |  | 1. Item 16 " the potential awkwardness correctly" was modified as " the potential awkwardness and inconvenience correctly".  2. Item 25 " When injecting insulin" was modified as " When injecting insulin in a standard way".  3. Item 29 " I will adjust my insulin dose according to my blood sugar level under the guidance of my doctor" was modified as "Under the guidance of my doctor, I will adjust the insulin dose according to diet, exercise and blood sugar level".  4. Item 34 " I know how to deal with hypoglycemic symptoms" was modified as " I know how to deal with hypoglycemic symptoms such as palpitations, sweating, shaking hands, and hunger". |
| **Results** |  | Finally, the scale comprised of 44 items and 4 dimensions as follows. |

**Item pool comprised of 44 items**

| Knowledge | Item 1: Diabetes is a disease caused by insulin deficiency and/or utilization disorder.  Item 2: Insulin is a physiological hormone secreted by the body that directly lowers blood sugar.  Item 3: Insulin therapy is one of the effective ways to lower blood sugar.  Item 4: The initiation of insulin therapy is determined by a combination of factors such as my blood sugar level, pancreatic function, complications, and other factors, and does not represent the severity of the disease  Item 5: The use of insulin is the natural progression of diabetes and does not imply the aggravation of the disease.  Item 6: Injecting insulin indicates failure of pre-insulin treatment.  Item 7: Insulin can prevent damage to liver and kidney function  Item 8: Insulin can improve pancreatic function  Item 9: Insulin therapy is an exogenous supplement or replacement for insulin deficiency in the body, which does not cause dependence or addiction in the body.  Item 10: I know the name, dosage, and type of insulin I use.  Item 11: I keep an eye on the expiration date of the insulin I use.  Item 12: I know that unopened insulin should be refrigerated at 2-8℃, avoiding freezing and direct sunlight.  Item 13: I know that insulin that has been opened does not need to be refrigerated at 2-8℃, but can be stored at room temperature below 30 ℃ and must be used up within 4 weeks.  Item 14: I know that different insulin cannot be freely converted between them. |
| --- | --- |
| Attitude | Item 15: I believe that the insulin prescribed by the doctor can prevent or delay the occurrence of complications, such as diabetic kidney disease and diabetic eye disease, etc.  Item 16: When using insulin outside, I can effectively avoid or view the potential awkwardness and inconvenience correctly.  Item 17: When I inject insulin, I worry that others will know that I have diabetes.  Item 18: When I feel good, I can reduce my insulin dose at will.  Item 19: When I feel good, I can stop insulin.  Item 20: When blood sugar control reaches the target value, I can reduce insulin dosage.  Item 21: When blood sugar control reaches the target value, I can stop insulin.  Item 22: I think it is not a big deal to reduce, miss and stop insulin privately.  Item 23: I will look upon insulin related information on the Internet objectively and rationally.  Item 24: Injecting insulin is painful.  Item 26: Insulin means I have to give up activities I enjoy.  Item 27: When using insulin, there is no need to control diet and exercise.  Item 28: When injecting insulin in a standard way, there is basically no pain, just like being bitten by a mosquito.  Item 29: Insulin will make others perceive greater sickness. |
| Practice | Item 30: I will purchase insulin according to the doctor's prescription.  Item 31: Under the guidance of my doctor, I will adjust the insulin dose according to diet, exercise and blood sugar level.  Item 32: When I have questions about insulin, I will consult medical staff (doctors, nurses, pharmacists).  Item 33: When I forget to inject insulin, I know how to deal with it.  Item 34: During the use of insulin, if I experience any discomfort, I will promptly report it to medical staff.  Item 35: I will go to the hospital for regular follow-up and adjust the insulin dosage. |
| Skill | Item 36: I'll rotate the injection site each time.  Item 37: I know how to deal with hypoglycemic symptoms such as palpitations, sweating, shaking hands, and hunger.  Item 38: I change my insulin needles each time.  Item 39: I will inject insulin at the time recommended by my doctor (such as half an hour before meals, immediately before meals, before bedtime, etc.)  Item 40: I will check the insulin injection site each time, avoiding areas with sunken skin, hard knots, and bruises.  Item 41: I will keep the needle under the skin for at least 10 seconds after insulin injection before pulling it out to prevent drug leakage.  Item 42: Before injecting medium acting insulin and premixed insulin, I will mix them well.  Item 43: I exhaust gas before injecting insulin to ensure accurate dosage  .  Item 44: Before using unopened insulin, I rewarm it before using it. |
