# Supplementary material for: Development and validation of a Chinese insulin medication literacy scale for patients with diabetes mellitus
Source: Front Pharmacol. 2025 Apr 2;16:1477050. doi: 10.3389/fphar.2025.1477050 (PMC11999841; doi:10.3389/fphar.2025.1477050)
Supplement: Supplementary file 11 [file Supplementaryfile4.docx]

**Supplementary file 4** Independent t-test between high score group and low score group

| Item | *t* | *sig(bilateral)* |
| --- | --- | --- |
| K1 | 12.219 | 0.000^**^ |
| K2 | 13.279 | 0.000^**^ |
| K3 | 10.139 | 0.000^**^ |
| K4 | 10.533 | 0.000^**^ |
| K5 | 13.849 | 0.000^**^ |
| K6 | 10.894 | 0.000^**^ |
| K7 | 9.808 | 0.000^**^ |
| K8 | 8.831 | 0.000^**^ |
| K9 | 12.761 | 0.000^**^ |
| K10 | 10.872 | 0.000^**^ |
| A1 | 7.895 | 0.000^**^ |
| A2 | 11.895 | 0.000^**^ |
| A3 | 9.297 | 0.000^**^ |
| A4 | 8.899 | 0.000^**^ |
| A5 | 6.037 | 0.000^**^ |
| A6 | 9.174 | 0.000^**^ |
| A7 | 9.846 | 0.000^**^ |
| A8 | 8.664 | 0.000^**^ |
| A9 | 9.318 | 0.000^**^ |
| A10 | 4.778 | 0.000^**^ |
| A11 | 10.387 | 0.000^**^ |
| A12 | -1.609 | 0.110 |
| P1 | 6.836 | 0.000^**^ |
| P2 | 9.850 | 0.000^**^ |
| P3 | 9.974 | 0.000^**^ |
| P4 | 11.898 | 0.000^**^ |
| P5 | 13.654 | 0.000^**^ |
| P6 | 12.185 | 0.000^**^ |
| P7 | 10.403 | 0.000^**^ |
| S1 | 10.391 | 0.000^**^ |
| S2 | 13.654 | 0.000^**^ |
| S3 | 13.727 | 0.000^**^ |
| S4 | 8.402 | 0.000^**^ |
| S5 | 9.159 | 0.000^**^ |
| S6 | 5.899 | 0.000^**^ |
| S7 | 12.045 | 0.000^**^ |
| S8 | 10.943 | 0.000^**^ |

Note: K is short for knowledge; A is short for attitude; P is short for practice; S is short for skill. ** *P*-value < 0.001
