# Supplementary material for: Development and validation of a Chinese insulin medication literacy scale for patients with diabetes mellitus
Source: Front Pharmacol. 2025 Apr 2;16:1477050. doi: 10.3389/fphar.2025.1477050 (PMC11999841; doi:10.3389/fphar.2025.1477050)
Supplement: Supplementary file 12 [file Supplementaryfile12.docx]

**Supplementary file 12:**

Final scale

| Domain |  | Item | Option |
| --- | --- | --- | --- |
| Knowledge | K1 | Diabetes is a disease caused by insulin deficiency and/or utilization disorder. | A. totally agree B. agree C. not sure D. disagree E. totally disagree |
|  | K2 | Insulin is the only physiological hormone secreted by the body that directly lowers blood sugar. | A. totally agree B. agree C. not sure D. disagree E. totally disagree |
|  | K3 | Insulin therapy is one of the effective ways to lower blood sugar. | A. totally agree B. agree C. not sure D. disagree E. totally disagree |
|  | K4 | The initiation of insulin therapy is determined by a combination of factors such as my blood sugar level, pancreatic function, complications, and other factors, and does not fully represent the severity of the disease. | A. totally agree B. agree C. not sure D. disagree E. totally disagree |
|  | K5 | Insulin therapy is an exogenous supplement or replacement for insulin deficiency in the body, which does not cause dependence or addiction in the body. | A. totally agree B. agree C. not sure D. disagree E. totally disagree |
|  | K6 | I know the name, dosage, and type of insulin I use. | A. right B. wrong |
|  | K7 | I keep an eye on the expiration date of the insulin I use. | A. right B. wrong |
|  | K8 | I know that unopened insulin should be refrigerated at 2-8 ° C, avoiding freezing and direct sunlight. | A. right B. wrong |
|  | K9 | I know that insulin that has been opened does not need to be refrigerated at 2-8 ° C, but can be stored at room temperature below 30 ℃ and must be used up within 4 weeks. | A. right B. wrong |
|  | K10 | I know that there is no arbitrary conversion between medium/long-acting insulin and short/rapid acting insulin. | A. right B. wrong |
| Attitude | A1 | I believe that the insulin prescribed by the doctor can help me control my blood sugar, so as to prevent or delay the occurrence of complications, such as diabetic kidney disease and diabetic eye disease, etc. | A. totally agree B. agree C. not sure D. disagree E. totally disagree |
|  | A2 | When using insulin outside, I can effectively avoid or view the potential awkwardness and inconvenience correctly. | A. totally agree B. agree C. not sure D. disagree E. totally disagree |
|  | A3 | When I inject insulin, I worry that others will know that I have diabetes. | A. totally agree B. agree C. not sure D. disagree E. totally disagree |
|  | A4 | When I feel good, I can reduce my insulin dose at will. | A. totally agree B. agree C. not sure D. disagree E. totally disagree |
|  | A5 | When I feel good, I can stop insulin. | A. totally agree B. agree C. not sure D. disagree E. totally disagree |
|  | A6 | When blood sugar control reaches the target value, I can reduce insulin dosage. | A. totally agree B. agree C. not sure D. disagree E. totally disagree |
|  | A7 | When blood sugar control reaches the target value, I can stop insulin. | A. totally agree B. agree C. not sure D. disagree E. totally disagree |
|  | A8 | I think it is not a big deal to reduce, miss and stop insulin privately. | A. totally agree B. agree C. not sure D. disagree E. totally disagree |
|  | A9 | I will look upon insulin related information on the Internet objectively and rationally. | A. totally agree B. agree C. not sure D. disagree E. totally disagree |
|  | A10 | When using insulin, there is no need to control diet and exercise. | A. totally agree B. agree C. not sure D. disagree E. totally disagree |
|  | A11 | When injecting insulin in a standard way, there is basically no pain, just like being bitten by a mosquito. | A. totally agree B. agree C. not sure D. disagree E. totally disagree |
| Practice | P1 | I will purchase insulin according to the doctor's prescription. | A. always B. often C. sometimes D. seldom E. never |
|  | P2 | I will monitor my blood sugar under the guidance of my doctor. | A. always B. often C. sometimes D. seldom E. never |
|  | P3 | Under the guidance of my doctor, I will adjust the insulin dose according to diet, exercise and blood sugar level. | A. always B. often C. sometimes D. seldom E. never |
|  | P4 | When I have questions about insulin, I will consult medical staff (doctors, nurses, pharmacists). | A. always B. often C. sometimes D. seldom E. never |
|  | P5 | When I forget to inject insulin, I know how to deal with it. | A. right B. wrong |
|  | P6 | During the use of insulin, if I experience any discomfort, I will promptly report it to medical staff. | A. always B. often C. sometimes D. seldom E. never |
|  | P7 | I will go to the hospital for regular follow-up and adjust the insulin dosage. | A. always B. often C. sometimes D. seldom E. never |
| Skill | S1 | I'll rotate the injection site each time. | A. always B. often C. sometimes D. seldom E. never |
|  | S2 | I know how to deal with hypoglycemic symptoms such as palpitations, sweating, shaking hands, and hunger. | A. right B. wrong |
|  | S3 | I change my insulin needles each time | A. always B. often C. sometimes D. seldom E. never |
|  | S4 | I will inject insulin at the time recommended by my doctor (such as half an hour before meals, immediately before meals, before bedtime, etc.). | A. always B. often C. sometimes D. seldom E. never |
|  | S5 | I will check the insulin injection site each time, avoiding areas with sunken skin, hard knots, and bruises. | A. always B. often C. sometimes D. seldom E. never |
|  | S6 | I will keep the needle under the skin for at least 10 seconds after insulin injection before pulling it out to prevent drug leakage. | A. always B. often C. sometimes D. seldom E. never |
|  | S7 | I exhaust gas before injecting insulin to ensure accurate dosage. | A. always B. often C. sometimes D. seldom E. never |
|  | S8 | Before using unopened insulin, I rewarm it before using it. | A. always B. often C. sometimes D. seldom E. never |
